# Revealing the diversity of *Parasmittina* Osburn, 1952 (Bryozoa, Cheilostomatida) from the Southwest Atlantic: Species complexes, non-native and new species

**DOI:** 10.1371/journal.pone.0304347

**Published:** 2024-08-08

**Authors:** Jamile Farias, Leandro M. Vieira, Ana C. S. Almeida

**Affiliations:** 1 Programa de Pós-Graduação em Biodiversidade e Evolução, Instituto de Biologia, Universidade Federal da Bahia, Salvador, BA, Brazil; 2 Museu de História Natural da Bahia, Setor da Zoologia, Instituto de Biologia, Universidade Federal da Bahia, Salvador, BA, Brazil; 3 Laboratório de Estudos de Bryozoa–LAEBry, Departamento de Zoologia, Centro de Biociências, Universidade Federal de Pernambuco, Recife, PE, Brazil; 4 Scientific Associate of the Department of Life Sciences, Natural History Museum, London, United Kingdom; Wrocław University of Environmental and Life Sciences: Uniwersytet Przyrodniczy we Wroclawiu, POLAND

## Abstract

*Parasmittina* is the most representative cheilostome genus of the family Smittinidae, often reported in the fouling non-indigenous marine community. Here, we present a review of *Parasmittina* species reported in the Southwestern Atlantic including the characterization of one species from Argentina (*P*. *dubitata*) and ten from the Brazilian coast: *P*. *abrolhosensis*, *P*. *alba*, *P*. *bimucronata*, *P*. *ligulata* comb. nov., *P*. *longirostrata*, *P*. *pinctatae*, *P*. *serrula*, *P*. *simpulata*, *P*. *winstonae* and the new species *Parasmittina falciformis* sp. nov. The new species is characterized by a smooth distally primary orifice with 1–2 oral spines, large lyrula, serrated condyles with hooked tips, and two types of avicularia–small and subtriangular and large sublanceolate. This study does not recognize four species previous recorded in Brazil: reports of *P*. *betamorphaea* and *P*. *trispinosa* are now assigned to *P*. *pinctatae*; records of *P*. *munita* belong to *P*. *falciformis* sp. nov.; and reports of *P*. *spathulata* encompass at least two taxa, including *P*. *abrolhosensis* and *P*. *simpulata*. In this study, five species complexes (*P*. *alba*, *P*. *longirostrata*, *P*. *serrula*, *P*. *simpulata* and *P*. *winstonae*) were identified and require further investigations. While six species characterized here were first described based on specimens from the Southwestern Atlantic (*P*. *abrolhosensis*, *P*. *alba*, *P*. *dubitata*, P. *ligulata* comb. nov., *P*. *simpulata* and *P*. *falciformis* sp. nov.), the remaining species are mainly known from the Indo-Pacific. These taxa are here recognized as exotic (*P*. *longirostrata*) and cryptogenic (*P*. *bimucronata*, *P*. *pinctatae*, *P*. *serrula* and *P*. *winstonae*) in the studied area. Most of the non-native taxa are widespread along the Brazilian coast, growing on both artificial and natural surfaces, indicating that they are well-established in the area. As non-native bryozoans can negatively influence the environment, affecting human economic activities and beach usage, further studies on the fauna presented here are suggested to determine the origin of these taxa and help prevent bioinvasion events along the SW Atlantic.

## Introduction

Smittinidae Levinsen, 1909 is among the most diverse families of cheilostome bryozoans, currently comprising 20 genera distributed worldwide, six of them reported from the Southwestern (SW) Atlantic: *Amynaskolia* Figuerola, Gordon & Cristobo, 2018; *Hemismittoide*a Soule & Soule, 1973; *Parasmittina* Osburn, 1952; *Pleurocodonellina* Soule & Soule, 1973; *Smittina* Norman, 1903 and *Smittoidea* Osburn, 1952 [1]. Among these genera, *Parasmittina* stands out for being the richest one, with more than 150 living species [1], representing a highly successful group in reefs habitats across all oceans (e.g., [2–4]). Most *Parasmittina* species have been reported from the Pacific, with more than 50 living species [2, 5, 6]. More recently, new species from other area, including the SW Atlantic, have been described (e.g., [7–13]).

Like most cheilostome bryozoans, smittinids have short-living, non-planktotrophic larvae with limited active dispersal over long distances (e.g., [14, 15]). However, certain taxa, including *Parasmittina* species, have been reported far from their expected natural geographic distribution, indicating association with bioinvasion events (e.g., [4, 9, 16, 17]). Therefore, the accurate taxonomic identification of these taxa represents the first step in detecting and tracking potential invasions (e.g., [14, 15]). However, the taxonomy of several Smittinidae taxa may be puzzling, sometimes leading to generic misassignments, notably in the case of *Parasmittina* (e.g., [4, 18–20]). Studies on smittinids from Brazil, in the SW Atlantic, have resulted in morphological redefinitions of *Hemismittoidea*, *Smittoidea*, and *Parasmittina* [4]. These redifinitions facilitate the differentiation between these genera, indicating that *Parasmittina* comprises smittinids with an imperforate frontal shield, none to many spines, and avicularia with variable morphology and sizes placed proximolateral or distolateral to the orifice [4]. Similar to genus delimitation, species distinctions can be also problematic, particularly when only colonial fragments are available. These specimens may obscure variations related to colonial development in different ontogenetic stages, making it challenging to differentiate distinct morphospecies [4, 9, 21].

Eleven species of *Parasmittina* have been reported from the SW Atlantic: *P*. *abrolhosensis* Ramalho, Taylor, & Moraes in Ramalho *et al*., 2018, *P*. *alba* Ramalho, Muricy & Taylor, 2011, *P*. *amazonensis* Ramalho & Moraes in Ramalho *et al*. 2021, *P*. *betamorphaea* Winston, 2005, *P*. *distincta* Ramalho, Taylor, & Moraes in Ramalho *et al*., 2018, *P*. *dubitata* Hayward, 1980, *P*. *loxoides* Winston, Vieira & Woollacott, 2014, *P*. *munita* (Hincks, 1884), *P*. *simpulata* Winston, Vieira & Woollacott, 2014, *P*. *spathulata* (Smitt, 1873) and *P*. *trispinosa* (Johnston, 1838) [22]. Among these, *P*. *munita*, *P*. *spathulata* and *P*. *trispinosa* have been assigned to be part of the *P*. *trispinosa* complex [5, 23, 24]. Although first described based on specimens from the Northeastern Atlantic, *P*. *trispinosa* has been historically reported in different oceans with different morphologies attributed to intraspecific variations (e.g., *P*. *trispinosa* var. *loxa* and *P*. *trispinosa* var. *spathulata*). However, Soule & Soule [5] reviewed historical records of *P*. *trispinosa* using scanning electron microscopy (SEM), assigning them to 14 species, eight of which were described as new species. The need for taxonomic reviews in some of these taxa was previously indicated by Vieira *et al*. [25], Almeida *et al*. [26] and Farias *et al*. [4]. In this context, here we reexamined SW Atlantic specimens assigned to the genus *Parasmittina*, providing morphological characterization and assessing the exotic status of 12 species, including the description of a new species. Additionally, a tabular identification key with almost all species of the genus is provided.

## Material and methods

All necessary permits for the new samples were obtained for the described field studies in Brazil (collecting permit number 47108 SISBIO/Instituto Chico Mendes de Conservação da Biodiversidade). The new samples in reported localities do not include protected areas and did not involve endangered or protected species. Permissions from all museums were obtained to access and study their collections.

Type and non-type specimens studied here are deposited in the Bryozoa collections of the Setor de Zoologia do Museu de História Natural da Bahia, Universidade Federal da Bahia, Salvador, Brazil (UFBA); Departamento de Zoologia, Universidade Federal de Pernambuco, Recife, Brazil (UFPE); Museu de Zoologia, Universidade de São Paulo (MZUSP); Smithsonian Institution National Museum of Natural History, Washington D.C., USA (USNM); Natural History Museum, London, United Kingdom (NHMUK); and Museum für Naturkunde, Berlin, Germany (MFN). The specimen from Argentina was analyzed based on scanning electron microscope (SEM) micrographs provided by Juan López Gappa.

Non-type specimens were first washed in sodium hypochlorite and then immersed in water for surface cleaning using a thin brush. These specimens were air-dried before examination under a stereoscopic microscope for the description of external morphology and delimitation of morphotypes. Selected specimens of each morphotype were mounted on stubs, coated with gold, and analyzed with a SEM JEOL JSM–6390LV at the Centro de Pesquisa Gonçalo Moniz, FIOCRUZ, Bahia, Brazil. Some unbleached specimens deposited at the NHMUK and USNM were examined using a SEM equipped with a low-vacuum chamber and back-scattered electron detector, using a SEM LEO 1455–VP and PhilipsXL30, respectively.

Measurements were obtained from digital SEM images using ImageJ^®^ software [27]. They are presented based on the following characters: autozooid length (ZL), autozooid width (ZW), primary orifice length (OL), primary orifice width (OW), avicularia length (AvL), avicularia width (AvW), ovicell length (OvL), and ovicell width (OvW) (S1 Table). The measurements are represented in the descriptions as minimum–median–maximum, long with the number of measurements (n) and standard deviation (SD), all in millimeters (mm). The taxonomy follows Martha *et al*. [28].

The determination of the native/exotic status of the studied species was conducted following the criteria described by Chapman & Carlton [29, 30] and adapted for Bryozoa by Miranda *et al*. [15] and Xavier *et al*. [31].

Fig 19 was created in Adobe Photoshop (https://photoshop.adobe.com/) using a basemap available in Canva (https://www.canva.com/) under CC BY 4.0 license.

### Nomenclatural acts

The electronic edition of this article conforms to the requirements of the amended International Code of Zoological Nomenclature, and hence the new names contained herein are available under that Code from the electronic edition of this article. This published work and the nomenclatural acts it contains have been registered in ZooBank, the online registration system for the ICZN. The ZooBank LSIDs (Life Science Identifiers) can be resolved, and the associated information viewed through any standard web browser by appending the LSID to the prefix “http://zoobank.org/”. The LSID for this publication is: urn:lsid:zoobank.org:pub:FEC88DB2-877B-46E4-94C0-DEF4C8F449BD. The electronic edition of this work was published in a journal with an ISSN, and has been archived and is available from the following digital repositories: LOCKSS (added: Sep 10 2012 6:55PM UTC) [http://www.lockss.org]; PubMed Central (added: Sep 10 2012 6:55PM UTC) [http://www.ncbi.nlm.nih.gov/pmc].

## Results

### Order Cheilostomatida Busk, 1852

#### Family Smittinidae Levinsen, 1909

**Type genus.** *Smittina* Norman, 1903 (type species *Lepralia landsborovii* Johnston, 1847, by original designation).

**Remarks.** Characteristics of the primary orifice, presence of lyrula, avicularium, and presence of pseudopores on the frontal wall and ectooecium, serve as the main taxonomic criteria to distinguishing Smittinidae genera [2, 5, 9, 24, 32]. While most genera encompass fewer than ten species, *Parasmittina*, *Smittina*, and *Smittoidea* are considered highly diverse, with *Parasmittina* and *Smittina* comprising more than 100 known species each [1]. *Parasmittina* and *Smittoidea* share morphological similarities, leading to misinterpretations regarding diagnostic features and the generic placement of several taxa [4, 18, 19, 20, 21]. Therefore, a taxonomic review of both *Parasmittina* and *Smittoidea* is strongly recommended [4, 9, 17].

### Genus *Parasmittina* Osburn, 1952

#### Type species

*Lepralia jeffreysi* Norman, 1876, by original designation.

#### Diagnosis

Autozooids with imperforate frontal wall, except for marginal pores. Primary orifice with smooth or beaded distal margin, oral spines usually present, lyrula of variable size and condyles with distinct profiles. Secondary orifice with variable development, sometimes with a proximal pseudosinus. Adventitious avicularia formed from a marginal pore [for example, as in *P*. *jeffreysi* (acc. Winston & Hayward [33]), *P*. *nitida* and *P*. *winstonae*], or sometimes, from a latero-oral pore (for example, as in *P*. *pinctatae*, *P*. *simpulata* and *P*. *falciformis* sp. nov.); adventitious avicularia with diverse shapes and positions (frontal, latero-oral or marginal), but never suboral, unless if displaced by the secondary calcification. Vicarious avicularia may also be present. Ovicells hyperstomial, initially prominent and becoming immersed with secondary calcification; ectooecium with pseudopores (modified from Osburn [24]).

#### Remarks

Historically, the taxonomic identification of *Parasmittina* was primarily based on the morphology and position of the adventitious avicularia [3, 7, 23, 24, 34–37]. Soule & Soule [2, 5] made extensive taxonomic studies on smitinids, particularly *Parasmittina*, revealing that a combination of morphological characters is necessary for confident identification. However, new species of *Parasmittina* have been distinguished from congeners primarily based on avicularia morphology (e.g., [7, 10]) or were described based on colonies fragments (i.e., [11, 12, 38]) that may not encompass intraspecific variations of these taxa, especially regarding avicularia development [2, 4, 5, 9, 17, 21, 39].

Thus, in this study, we attempt to analyze colonies at various astogenetic stages, comparing them with all congeners reported worldwide and using both morphological and morphometric data. Diagnostic features were based mainly on the primary orifice (distal margin ornamentation, number of spines, lyrula width, and condyles morphology), avicularia (if adventitious and/or interzooidal, placement, orientation, size, and morphologies), and ooecia (number of pseudopores, and secondary calcification).

#### *Parasmittina abrolhosensis* Ramalho, Taylor, & Moraes in Ramalho *et al*., 2018

**Fig 1:**
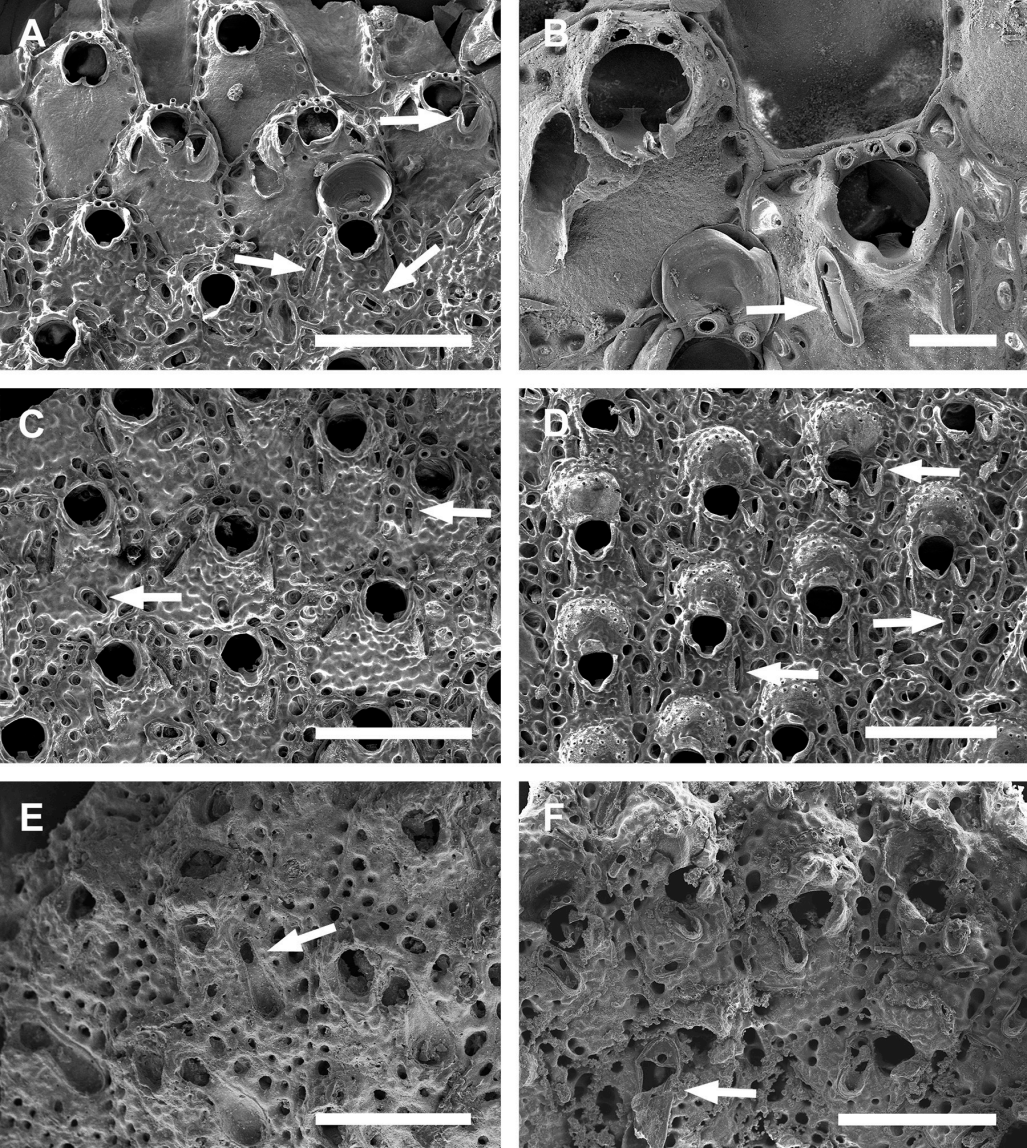
*Parasmittina abrolhosensis* Ramalho, Taylor, & Moraes in Ramalho *et al*., 2018. (A, C–D) UFBA 5004, (B) UFBA 2416, (E) UFBA 696, (F) UFBA 5008, Bahia, Brazil. Arrows indicating avicularia. (A) growing edge of the colony with small avicularia, (B) detail of primary orifice and small elongate avicularia, (C) autozooids with secondary calcification and small avicularia, (D) group of ovicelled zooids with small avicularia, (E) old autozooids with large spatulate avicularia, (F) ovicelled zooids with secondary calcification and large spatulate avicularia. Scale bars: A, C–F = 400 μm; B = 100 μm.

**Fig 2:**
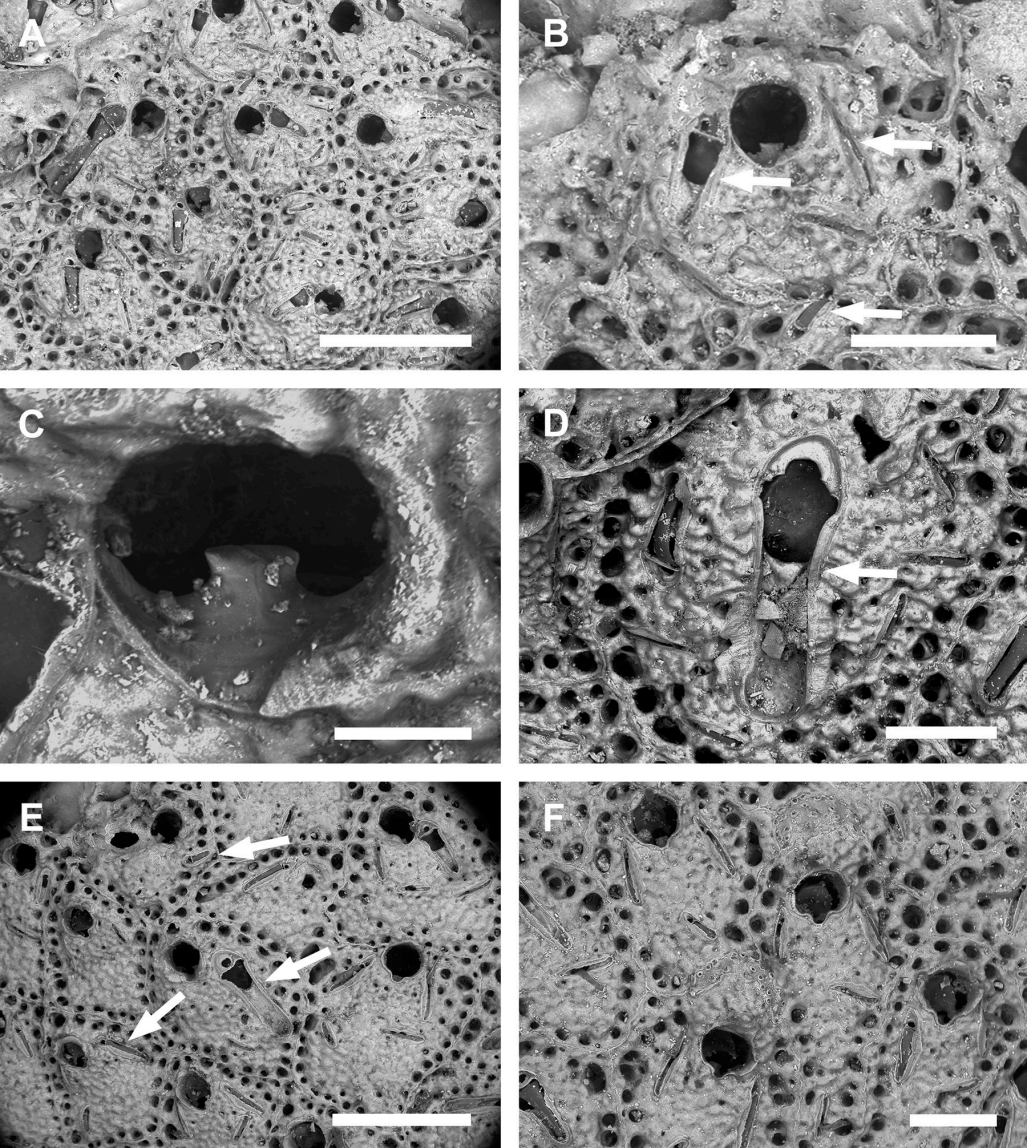
*Parasmittina abrolhosensis* Ramalho, Taylor, & Moraes in Ramalho *et al*., 2018. (A–D) USNM 8558, (E–F) USNM 8559, Bahia, Brazil. (A) group of autozooids, (B) detail of an autozooid showing small elongate and subtriangular and large spatulate avicularia, (C) detail of primary orifice, (D) detail of vicarious avicularia, (E) group of young autozooids with small and large avicularia, (F) group of ovicelled zooids. Scale bars: A, E = 500 μm; B, D, F = 200 μm; C = 50 μm.

(Figs 1, 2; Tables 1, 10)

**Table 1:** Morphometric data of *P*. *abrolhosensis*. Values given in millimeters and represented as minimum–median–maximum (number of zooidal measures). Avicularia abbreviations: Av1 = small elongate; Av2 = small subtriangular bulbous; Av3 = small subtriangular; Av4 = large spatulate; Av5 = vicarious spatulate.

Character				
	Ramalho *et al*. (2018) / MNRJ-Bry1359	Canu & Bassler (1928) / USNM 8558	Canu & Bassler (1928) / USNM 8559	Present study / UFBA 2877
**ZL**	0.312–0.396–0.471 (?)	0.369–0.513–0.582 (12)	0.398–0.568–0.749 (13)	0.338–0.418–0.606 (15)
**ZW**	0.280–0.308–0.360 (?)	0.276–0.370–0.463 (12)	0.273–0.416–0.538 (13)	0.237–0.318–0.411 (15)
**OL**	0.093–0.106–0.113 (?)	0.092–0.107–0.123 (6)	0.104–0.119–0.139 (7)	0.078–0.095–0.107 (12)
**OW**	0.075–0.084–0.099 (?)	0.082–0.094–0.107 (6)	0.093–0.104–0.125 (7)	0.084–0.096–0.109 (12)
**Av1L**	0.121–0.142–0.178 (?)	0.102–0.135–0.159 (7)	0.118–0.168–0.250 (15)	0.093–0.118–0.146 (15)
**Av1W**	-	0.006–0.009–0.013 (8)	0.009–0.019–0.029 (15)	0.011–0.017–0.022 (15)
**Av2L**	-	-	-	0.083–0.104–0.123 (10)
**Av2W**	-	-	-	0.042–0.051–0.056 (10)
**Av3L**	0.079–0.090–0.106 (?)	0.073–0.096–0.112 (15)	0.073–0.101–0.113 (10)	0.073–0.090–0.102 (15)
**Av3W**	-	0.008–0.014–0.019 (15)	0.011–0.015–0.024 (10)	0.024–0.029–0.033 (15)
**Av4L**	-	0.365–0.373–0.381 (2)	0.400 (1)	0.201 (1)
**Av4W**	-	0.073–0.075–0.077 (2)	0.073 (1)	0.048 (1)
**Av5L**	-	0.521 (1)	-	-
**Av5W**	-	0.141 (1)	-	-
**OvL**	0.170–0.183–0.204 (?)	0.157–0.174–0.200 (3)	0.172–0.185–0.209 (4)	0.148–0.161–0.193 (15)
**OvW**	0.219–0.236–253 (?)	0.167–0.192–0.230 (3)	0.218–0.244–0.273 (4)	0.203–0.214–0.229 (15)

*Parasmittina abrolhosensis* Ramalho, Taylor, & Moraes in Ramalho *et al*., 2018: 165, Figs 3H, 5A–D [11].

*Smittina areolata*: Canu & Bassler 1928: 30, plate 6 Fig 4 [40]. Non *Smittina areolata* Canu & Bassler, 1927: 23 [41].

**Fig 3:**
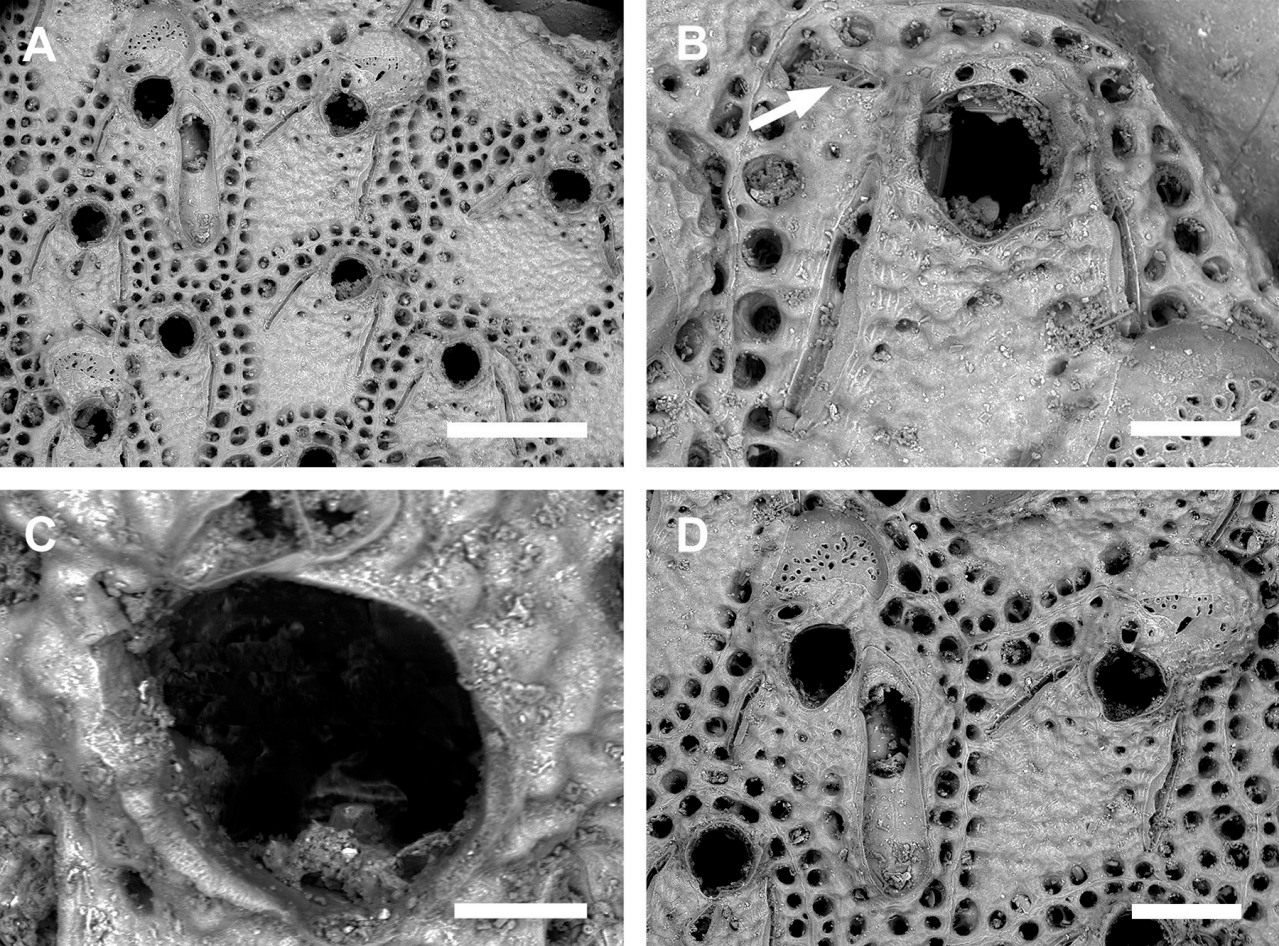
*Parasmittina areolata* (Canu & Bassler, 1927) (A–D) USNM 8443, holotype, Hawaii, USA. Arrows indicating avicularia. (A) group of autozooids and ovicelled zooids, (B) detail of autozooid with small avicularia, (C) detail of primary orifice, (D) detail of ovicelled zooids with small and large avicularia. Scale bars: A = 400 μm; B = 100 μm; C = 50 μm.

*Smittina trispinosa spathulata*: Canu & Bassler 1928: 29, plate 6, Fig 3 [40]; *Parasmittina spathulata*: Vieira *et al*. 2008: 27 (in part) [25], Almeida *et al*. 2015: 4 (in part) [26]. Non *Escharella jacotini* var. *spathulata* Smitt, 1873: 60 [42].

#### Material examined

UFPE 813–815; UFBA 2414–2416, 2855–2859, 2861–2862, 2879–2880, 2882–2884, 2886–2888, 3128, 5004–5007, Recife dos Cascos, Baia de Todos os Santos, Bahia, Brazil, 13–21 m, coll. 2016–2017; UFBA 696, 3120, Costa do Dendê, Bahia, Brazil, coll. 2002; UFBA 5008, Camaçari, Bahia, Brazil, 50 m, coll. 07/2004; UFPE 652–661, 2788–2797, Bacia Potiguar, Rio Grande do Norte, coll. 2009–2010; USNM 8558, 8559, *Smittina trispinosa spathulata* and *Smittina areolata*, respectively, F. Canu & R. Bassler det., Bahia, Brazil, 49 m, coll. 1876.

#### Description

Colony encrusting. Autozooids (Fig 1A) polygonal, almost rhombic, limited by slightly raised lateral walls, surrounded by a single row of 19–23 marginal pores (0.009–0.025–0.042 mm in diameter; n = 30; SD = 0.008 mm). Frontal wall initially smooth in very young zooids (Fig 1A, top; 1B), becoming rough, rugose, and with nodular calcification in older zooids (Fig 1C and 1D). Primary orifice (Fig 1B) elliptical, distal margin smooth, with 1–3 oral spines, lyrula narrow (0.012–0.021–0025 mm wide; n = 30; SD = 0.004 mm); a pair of robust and down-curved hooked condyles with serrated margins. Secondary orifice (Fig 1B and 1C) pear-shaped, forming 2–4 lateral flaps in autozooids, often with in a shallow U-shaped pseudosinus, more pronounced in older or ovicelled zooids (Fig 1D and 1E). Adventitious avicularia variable in shapes and sizes (Fig 1A and 1D), with four morphologies: (1) small, narrow and elongate avicularia (Figs 1C and 1E, 2B and 2E) present at one or both side of the orifice, unequal in size if paired, placed below to the secondary orifice, proximally oriented, rostrum narrow and slightly curved, smooth to slightly corrugated at its lateral margins, rounded tip, palate occupying about half of the rostrum length and oblong foramen; (2) small, subtriangular, bulbous avicularia (Fig 1A and 1D), placed laterally at one side of the orifice, proximally oriented, rostrum subtriangular, pointed tip, palate narrow, reaching twice its initial size with colonial development, leaving a sublanceolate profile; (3) small, narrow and subtriangular to elliptic avicularia (Figs 1C and 1E, 1F, 2B and 2E), placed in zooidal margins and/or in the frontal wall, with smooth lateral margins, rounded tip, palate narrow and elliptical foramen; (4) large spatulate avicularia, more common in older zooids, placed proximolaterally below the orifice, oriented proximally, with rounded distal edge, palate broad, foramen subtriangular, elongate rostrum, with smooth margins and crossbar complete, with 3–4 pseudopores adjacent to the avicularia. (Figs 1F and 2A, 2E); vicarious avicularia (Fig 2D) with the same morphology as the large spatulate avicularia occasionally present. Ovicells (Figs 1D and 2E) globose, initially prominent (Fig 1D) and densely surrounded by adjacent frontal wall in older colonies (Figs 1F and 2F); ectooecium with 18–25 small pseudopores (0.003–0.005–0.008 mm in diameter; n = 30; SD = 0.001 mm), mainly placed at the distal region of the ectooecium surface.

#### Remarks

Canu & Bassler [40] studied specimens from northeastern Brazil (Bahia) and attributed them to two species, *P*. *areolata* (Canu & Bassler, 1927) [41] and *P*. *spathulata* (Smitt, 1873). Following Canu & Bassler [40], Vieira *et al*. [25] and Almeida *et al*. [26] attributed other Brazilian specimens to *P*. *spathulata* (Smitt, 1873). These *P*. *areolata* and *P*. *spatulhata* records, as well as new studied specimens from the same region, are here attributed to *P*. *abrolhosensis*.

*Parasmittina areolata* was originally described by Canu & Bassler [41] based on specimens from Hawaii, Pacific Ocean. However, it has been reported in Brazilian coast and in the Caribbean [3, 37, 40]. Here we analyzed the type of *P*. *areolata* (USNM 8443), *Smittina areolata*, holotype, F. Canu & R. Bassler det., Molokai, Hawaii, 142–406 m, coll. 1902, Fig 3A–3D), revealing that this species is not conspecific with the Brazilian material. *Parasmittina areolata* has a double row of areolar pores (single in specimens from Brazil), low secondary orifice (with lateral flaps in Brazilian material) and ectooecium centrally perforated (distally perforated in specimens from Brazil). The morphologically related species *P*. *spathulata* is distinct from the Brazilian material in having a broader lyrula (acc. Winston [7]). Specimens from the Caribbean previously attributed to *P*. *areolata* by Winston [37] were reassigned to *P*. *luteoserrula* Winston & Jackson, 2021 (see Winston & Jackson [13]), distinct from Brazilian specimens by having an orifice with a serrated distal margin, and a large avicularium with a coarsely serrated margin.

**Fig 4:**
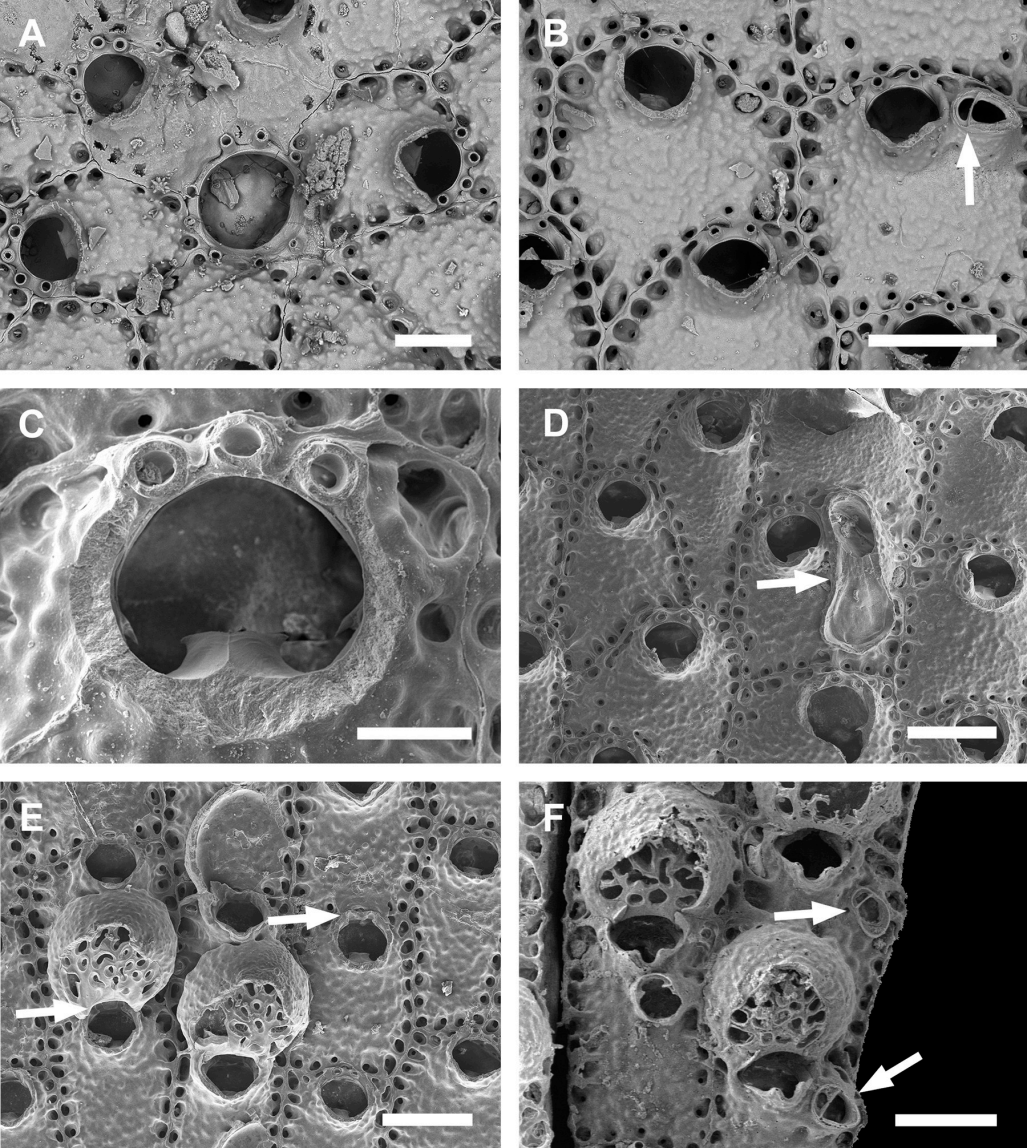
*Parasmittina alba* Ramalho, Muricy & Taylor, 2011. (A–B) UFBA 5059, Bahia, Brazil. Arrows indicating avicularia. (A) ancestrula and first zooids of the colony, (B) young autozooids showing small avicularium. (C–D) UFBA 5009, Bahia, Brazil. (C) detail of primary orifice, (D) group of young autozooids showing large avicularium, (E) UFBA 5010, Bahia, Brazil, colony with secondary calcification showing autozooids and ovicelled zooids, (F) UFPE 785, Espírito Santo, Brazil, detail of ovicelled zooids with small avicularia. Scale bars: A, B, D, E, F = 200 μm; C = 50 μm.

Specimens studied here shares most of the diagnostic characters provided by Ramalho *et al*. [11] in the description of *P*. *abrolhosensis*, including a primary orifice with distal margin smooth, lyrula narrow and robust and down-curved hooked condyles with serrated margins; secondary orifice with a U-shaped pseudosinus; avicularia with four morphologies: (1) small, narrow and elongate, (2) small, subtriangular and bulbous [not described by Ramalho *et al*. [11], but figured in 3H], (3) small, narrow and subtriangular to elliptic, and (4) large spatulate; and ovicells densely surrounded by adjacent frontal wall in older colonies, and with small pseudopores. However, specimens from Ramalho *et al*. [11] have 3–4 oral spines (1–3 in colonies studied here) and the large avicularium was not found as vicarious. Interestingly, the original description of *P*. *abrolhosensis* was based on three small colonial fragments inhabiting corals (acc. Ramalho *et al*. [11], Figs 3H, 4A–4D) whereas several colonies studied here are from artificial substrata and material from Canu & Bassler [40] are colonies with rough secondary calcification. Since the number of oral spines and occurrence of avicularia in *Parasmittina* are very variable in relation to colony development and habitat (e.g., [2, 4, 5, 9, 21, 39]), these differences are likely to be intraspecific variations of *P*. *abrolhosensis* rather than a different species.

#### Distribution

SW Atlantic: Brazil (Canu & Bassler [32]; Vieira *et al*. [25]; Almeida *et al*. [26]; Ramalho *et al*. [11]; present study).

### *Parasmittina alba* Ramalho, Muricy & Taylor, 2011

(Fig 4; Tables 2, 10)

**Table 2:** Morphometric data of *P*. *alba*, *P*. *lavela* and *P*. *betamorphea*. Values given in millimeters and represented as minimum–median–maximum (number of zooidal measures). Avicularia abbreviations: Av1 = small subtriangular; Av2 = small spatulate; Av3 = large spatulate.

Character					
	*P*. *alba /* Ramalho *et al*. (2011)	*P*. *alba* / Souto *et al*. (2016)	*P*. *alba /* UFBA 5010 (present study)	*P*. *lavela /* Soule & Soule (2002)	*P*. *betamorphea* / Winston (2005)
**ZL**	0.392–0.451–0.490 (?)	0.390–0.488–0.670 (24)	0.341–0.442–0.583 (15)	0.350–0.400 (?)	0.378–0.470–0.576 (6)
**ZW**	0.265–0.305–0.383 (?)	0.240–0.340–0.460 (24)	0.225–0.297–0.415 (15)	0.243–0.320 (?)	0.252–0.290–0.324 (6)
**OL**	**-**	0.076–0.082–0.101 (20)	0.074–0.085–0.098 (15)	**-**	0.072–0.080–0.090 (?)
**OW**	**-**	0.103–0.125–0.143 (20)	0.078–0.091–0.107 (15)	**-**	0.090–0.100–0.108 (?)
**Av1L**	0.098–0.109–0.118 (?)	0.068–0.078–0.089 (10)	0.093 (1)	0.120–0.150 (?)	0.072–0.090–0.162 (6)
**Av1W**	0.069–0.083–0.098 (?)	0.037–0.047–0.054 (10)	0.041 (1)	**-**	0.045–0.050–0.054 (6)
**Av2L**	**-**	0.074–0.092–0.104 (9)	**-**	**-**	**-**
**Av2W**	**-**	0.051–0.056–0.063 (9)	–	**-**	**-**
**Av3L**	0.353 (?)	**-**	0.304–0.329–0.355 (2)	**-**	0.324–0.360–0.396 (6)
**Av3W**	0.137 (?)	**-**	0.074–0.080–0.085 (2)	**-**	0.180–0.190–0.198 (6)
**OvL**	0.274 (?)	0.235–0.265–0.297 (20)	0.228–0.245–0.276 (15)	**-**	0.180–0.200–0.216 (2)
**OvW**	0.323 (?)	0.233–0.267–0.300 (20)	0.260–0.275–0.296 (15)	0.225–0.260 (?)	0.180–0.200–0.216 (2)

*Parasmittina alba* Ramalho *et al*., 2011: 769, figures 2A–H [10]; Souto *et al*. 2016: 3, figures 3A–H [17].

? *Smittia trispinosa* var. *protecta* Thornely, 1905: 123 [43];? *Parasmittina lavela* Soule & Soule, 2002: 36 [5];? *Parasmittina betamorphaea* Winston, 2005: 58 [7]

? *Parasmittina protecta*: Harmelin *et al*. 2009: 169 (cum. syn.) [9].

#### Material examined

UFPE 785, Ilhas Rasas, Guarapari, Espírito Santo, Brazil, 11–15 m, coll. 27/03/2017; UFPE 786–795; UFBA 5009–5010, 5013, 5016, 5027, 5033, 5036, 5041, 5045, 5047, 5054, 5058, 5062, 5065, 5069, 5074, 5079, 5084, 5088, 5098–5099, 5103, 5108–5109, 5120–5121, 5123, 5141–5142, 5148, 5156, 5165–5166, 5172, 5175, 5178, 5189, 5194, 5202–5203, 5207, 5210, 5215, 5221, 5228, 5235, 5240, 5246, 5251, 5257, 5263–5264, 5273–5274, 5281–5182, 5285, 5290, 5303, 5355, 5425, 5448, Baia de Todos os Santos, Salvador, Bahia, Brazil, 3–5 m, coll. 2012, MZUSP 2937, Alcatrazes, São Sebastião, São Paulo, Brazil, coll, 06/10/2011.

#### Description

Colony encrusting. Ancestrula (Fig 4A) tatiform, with narrow marginal cryptocyst, 8 marginal spines; 4–5 orificial spines in first daughter zooid; second and third daughter zooids with 3–4 orifical spines. Autozooids (Fig 4B) subrectangular, limited by slightly raised lateral walls, surrounded by a single row of 20–27 marginal pores (0.010–0.019–0.032 mm in diameter; n = 30; SD = 0.005 mm). Frontal wall rugose, with small, rounded tubercles. Primary orifice (Fig 4C) elliptical, distal margin smooth, with 1–3 oral spines; lyrula broad (0.038–0.046–0.058 mm wide; n = 15; SD = 0.005 mm), but shallow and often with a median keel; condyles paired, thin, with smooth margins and down-curved hooked tips. Secondary orifice (Fig 4E and 4F) raised around the proximal margin of the primary orifice, formed from lateral flaps, resulting in a shallow V-shaped pseudosinus. Adventitious avicularia variable in shape and size (Fig 4B, 4D and 4F), with three morphologies: (1) small, narrow, and subtriangular avicularia (Fig 4B and 4F), rare in very young zooids, placed laterally at one side of the orifice, commonly proximally oriented, rostrum subtriangular, finely serrated at its lateral margins, pointed tip, palate narrow and subtriangular foramen; (2) small and spatulate avicularia with broad rostrum (Fig 4F), rare in young zooids, placed laterally at one side of the orifice, proximally oriented, rostrum subspatulate, a rounded distal edge, palate narrow and elliptical foramen; (3) large spatulate avicularia (Fig 4D), more common in older zooids, placed laterally at one side of the orifice, oriented proximally, with a rounded distal edge, palate broad, foramen elliptical, with smooth margins and a complete crossbar, with 2–3 pseudopores adjacent to the avicularia. Ovicells (Fig 4E and 4F) surrounded by the frontal wall of adjacent zooids, becoming immersed in older colonies; ooecium almost globular with a flattened frontal surface (0.007–0.018–0.045 mm in diameter; n = 30; SD = 0.007 mm), ectooecium with 18–26 funnel-shaped pseudopores that become merged and polygonal with increasing calcification.

#### Remarks

*Parasmittina alba* was described based on specimens from Rio de Janeiro, SE Brazil; it was characterized by having a granular frontal wall, condyles with hooked tips, large lyrula, 1–2 oral spines, secondary orifice with lateral projections and pseudosinus, avicularia latero-oral (small subtriangular or rounded), large (spatulate) and interzooidal (subtriangular), with only one avicularium occurring per zooid, and ectooecium with pseudopores [10]. Most of these morphological characters are also seen in colonies from SE and NE Brazil examined here, despite the presence of 1–3 orificial spines and the rare presence of interzooidal avicularia. Most of our examined material form small patches growing on ceramic experimental tile plates (specimens from northeastern Brazil; Fig 4A), but the larger specimens have been only observed growing on rhodoliths (specimens from southeastern Brazil; Fig 4F). The avicularia were often found in older part of the colonies (Fig 4F) when comparing with the growing zone with the new zooids (Fig 4B), suggesting the formation of the avicularia in later colony astogeny. Moreover, we found no morphometric differences in colonies studied here assigned and other records of *P*. *alba* (Table 2).

*Parasmittina alba* was reported as a non-indigenous species in Portugal, Northeastern Atlantic [17]. Souto *et al*. [17] noted slight variations between specimens from Rio de Janeiro (acc. Ramalho *et al*. [10]) and those from Portugal, including smaller ectooecial pseudopores related with more significant calcification, the occurrence of two avicularia in ovicelled zooids and absence of interzooidal avicularia. Additionally, Souto *et al*. [17] suggested that *P*. *alba* could be assigned as a junior synonym of *P*. *betamorphaea* Winston, 2005 [7] described from the United States (Northwestern Atlantic) since characters used to distinguish these taxa are very variable among colonies and zooids (i.e., number of avicularia, calcification of margins and rostrum of the avicularia). The Caribbean *P*. *lavela* Soule & Soule [5] also shares most morphological diagnostic features of *P*. *alba*. These taxa have a granular frontal calcification, primary orifice without distal denticles, with large lyrula and thin down-curved hooked condyles, secondary orifice with a short pseudosinus, and ooecium with merged pseudopores [5]. The only difference between *P*. *alba* and *P*. *lavela* is the presence of a single morphotype of avicularia in *P*. *lavela* (i.e., small subtriangular latero-oral avicularia), lacking all other types described to *P*. *alba*. Soule & Soule [9] stated that avicularia are not common in *P*. *lavela*, but only a tiny single specimen growing on algae on an anchor chain was examined [5], somewhat corroborating morphological variation and biological associations already reported [10, 17] and assigned here to *P*. *alba*. Avicularia are known to be very variable in Smittinidae according to colony development and habitat (e.g., [2, 4, 5, 9, 21, 39]). Avicularia morphology is quite variable in specimens of *P*. *alba* here examined. We suspect that *P*. *alba* and *P*. *betamorphea* may represent different astogenetic variations of *P*. *lavela*–specimens of *P*. *alba* comprising a stage with all types of avicularia, ovicells, and secondary calcification [10], whereas *P*. *betamorphea* could represent young colony that lacks ovicells and have few avicularia [7].

Zooidal measurements of specimens attributed to *P*. *alba* and *P*. *betamorphea* are like those of *P*. *lavela* (Table 2). In addition, a peculiar feature of the species (as already mentioned by Souto *et al*. [17]) is a notch on the distal margin of the primary orifice of ovicellate zooids–this notch is formed in the place where the oral spines were inserted (Fig 4E) [see Ramalho *et al*. [10] (Fig 2G) and Souto *et al*. [17] (Fig 3B)]. However, observing this notch in colonies of *P*. *lavela* and *P*. *betamorphea* was impossible. Ovicelled zooids of *P*. *lavela* had the distal margin of the primary orifice occluded by secondary calcification [5] and *P*. *betamorphea* lacks ovicells [7]. Thus, at least one species complex involving *P*. *alba*, *P*. *betamorphea*, and *P*. *lavela* can be recognized (Table 2).

Finally, another congener may be involved in this complex. Specimens from the Southeast and Northeast Atlantic, as well as from the Mediterranean attributed to *Parasmittina protecta* (Thornely, 1905) (acc. Harmelin *et al*. [9]) also share most morphological diagnostic features of *P*. *alba*, being only variable in avicularia morphotypes occurrence. However, *P*. *protecta* was first described with colonies from the Indian Ocean and the original description is brief, not including diagnostic features of the primary orifice (distal margin ornamentation, condyles, lyrula) and detailed characterization of avicularia (acc. Thornely [43]). Also, there is no redescription of type or topotype specimens, preventing us from reliably characterizing *P*. *protecta* from Thornely (1905). Additional studies including more specimens are required to identify whether *P*. *protecta* and *P*. *alba* belong to the same species.

#### Distribution

Northeastern Atlantic: Portugal [17]; SW Atlantic: Brazil [10] present study).

### *Parasmittina bimucronata* (Hincks, 1884)

**Fig 5:**
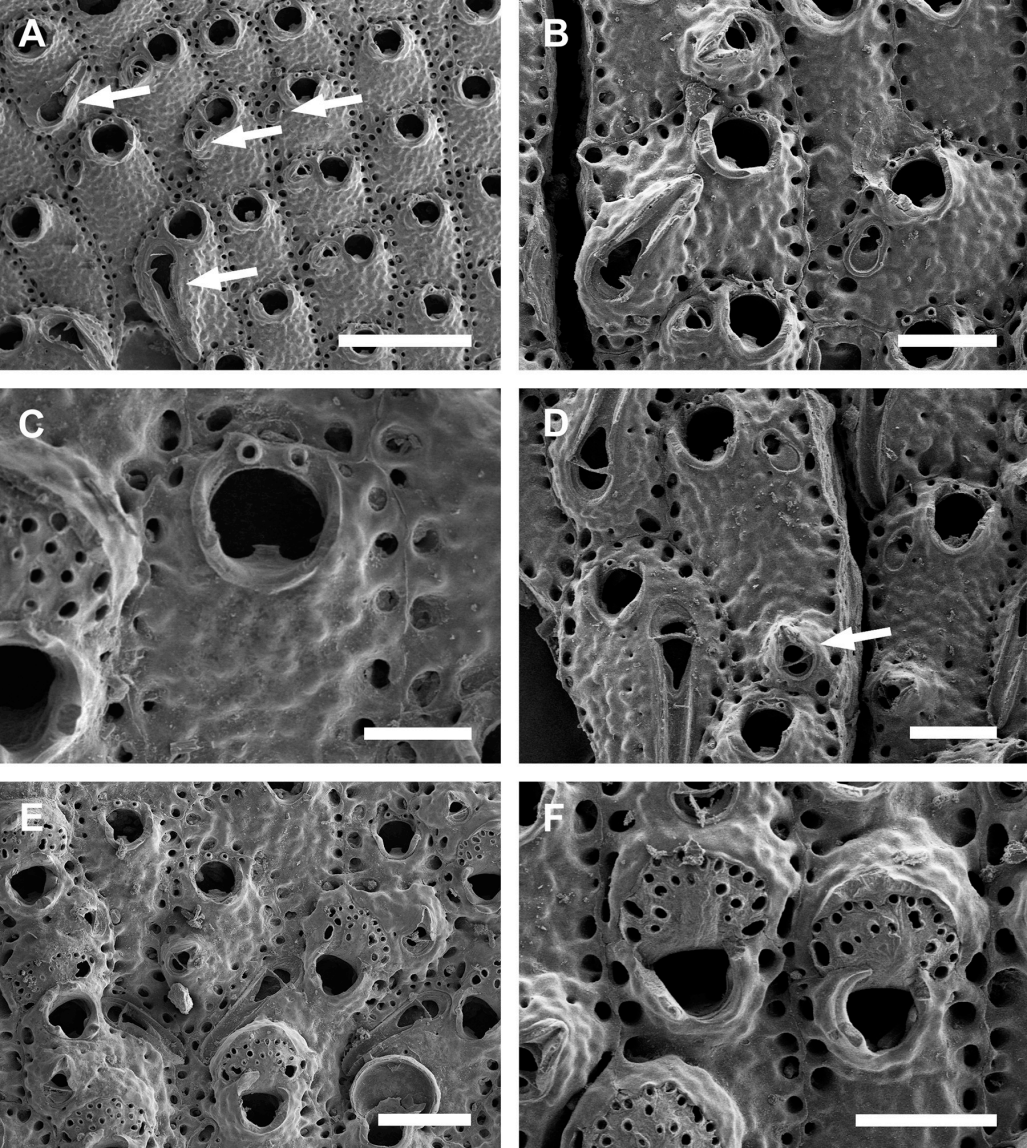
*Parasmittina bimucronata* (Hincks 1884a). (A–F) UFBA 946, Bahia, Brazil. Arrows indicating avicularia. (A) group of young autozooids with small and large latero-oral and large frontal avicularia, (B) detail of autozooids showing small latero-oral and large frontal avicularia, (C) detail of primary orifice, (D) autozooids with secondary calcification, (E) ovicelled zooids with secondary calcification, (F) detail of ovicells. Scale bars: A = 500 μm; B, D, E, F = 200 μm; C = 200 μm.

(Fig 5; Tables 3, 10)

**Table 3:** Morphometric data of *P*. *bimucronata*. Values given in millimeters and represented as minimum–median–maximum (number of zooidal measures). Avicularia abbreviations: Av1 = small subtriangular; Av2 = small oblong; Av3 = large sublanceolate.

Character	Reference / Specimen	
	Hayward & Parker (1994) / BMNH 1899.5.1.920^1^	Present study / UFBA 946
**ZL**	0.360 ± 004 (20)	0.224–0.398–0.695 (15)
**ZW**	0.230 ± 0.03 (20)	0.143–0.243–0.314 (15)
**OL**	0.080 ± 0.004 (20)	0.076–0.087–0.099 (15)
**OW**	0.090 ± 0.004 (20)	0.086–0.100–0.125 (15)
**AvZ1**	**-**	0.089–0.117–0.144 (15)
**AvW1**	**-**	0.033–0.052–0.069 (15)
**AvZ2**	**-**	0.100–0.105–0.111 (6)
**AvW2**	**-**	0.037–0.047–0.053 (6)
**AvZ3**	**-**	0.217–0.363–0.472 (15)
**AvW3**	**-**	0.034–0.054–0.073 (15)
**OvL**	**-**	0.150–0.194–0.210 (15)
**OvW**	**-**	0.216–0.255–0.293 (15)

**Fig 6:**
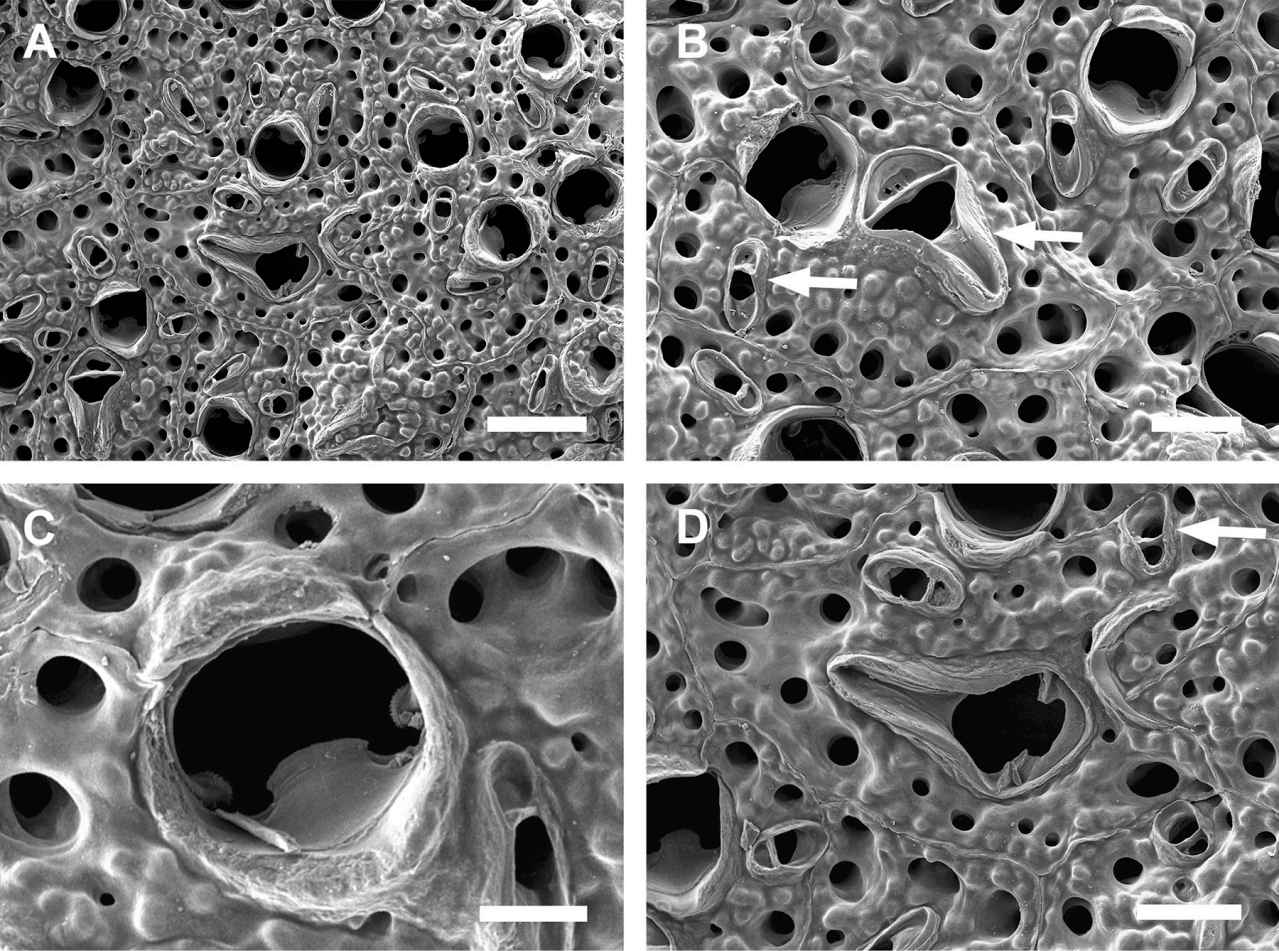
*Parasmittina dubitata* Hayward, 1980. (A–D) Uncatalogued specimen, Tierra del Fuego, Argentina. Arrows indicating avicularia. (A) group of autozooids showing small and large avicularia, (B) detail of autozooids with small oblong and large subtriangular avicularia, (C) detail of primary orifice, (D) detail of vicarious and small spatulate avicularia. Scale bars: A = 200 μm; B, D = 100 μm; C = 50 μm.

*Smittia trispinosa* form *bimucronata* Hincks, 1884a: 118, plate 13, figure 6 [44].

*Parasmittina bimucronata*: Hayward & Parker 1994: 70 [under remarks of *P*. *pectinata*] [43].

*Parasmittina glomerata*: Liu 2001: 618, plate 56, figures 5–6 [45]. Non *Smittia glomerata* Thornely, 1912: 152 [46].

#### Material examined

UFBA 946, Baía de Todos os Santos, Bahia, Brazil, coll. 1997; UFBA 3113, Costa do Dendê, Bahia, Brazil, coll. 2002.

#### Description

Colony encrusting. Autozooids (Fig 5A) subrectangular to polygonal, limited by slightly raised lateral walls, with a single row of 18–24 marginal pores (0.009–0.019–0.033 mm wide; n = 30; SD = 0.005 mm). Frontal wall rugose, with large, rounded tubercles. Primary orifice (Fig 5C) transversely elliptical, distal margin smooth, with 1–2 oral spines; lyrula narrow (0.019–0.025–0.032 mm wide; n = 15; SD = 0.004 mm), latero-proximal condyles triangular and thin. Secondary orifice initially low, becoming raised and forming lateral flap-shaped projections with increasing calcification, often with a shallow U-shaped pseudosinus (Fig 5D), partially obscuring primary orifice in latter astogeny, especially in ovicelled zooids, resulting in a deep U-shaped pseudosinus, but often not obscuring the lyrula (Fig 5E). Adventitious avicularia variable in shape and size (Fig 5A–5E), with three morphologies: (1) small, subtriangular, bulbous avicularia (Fig 5A and 5B), placed laterally at one side of the orifice, proximally oriented, rostrum subtriangular, corrugated at its lateral margins, pointed tip, palate narrow and subtriangular foramen; (2) small, oblong to spatulate avicularia (Fig 5A and 5B) single, common in younger zooids, placed below the secondary orifice, proximally oriented, rostrum subspatulate and straight, smooth at its lateral margins, rounded tip, palate occupying about half of the rostrum length and elliptical foramen; (3) large, sublanceolate avicularia (Fig 5A), placed laterally at one side of the orifice, oriented proximally, with acute rostrum, palate broad, foramen subtriangular, with finely serrated margins and crossbar complete, with 2–3 pseudopores adjacent to the avicularia. Large frontal avicularia (Fig 5A and 5B) occasionally present, with the same morphology as the latero-oral. Ovicells (Fig 5E–5F) wider than long, ooecium globular, becoming immersed in the frontal calcification with increasing calcification (Fig 5F); ectooecium with 20–27 pseudopores with slightly raised rim (0.004–0.011–0.020 mm wide; n = 30; SD = 0.004 mm).

#### Remarks

*Parasmittina bimucronata* is characterized by having the primary orifice smooth distally, 1–2 oral spines, thin and smooth condyles and lyrula occupying one-quarter of the orificial width; adventitious latero-oral avicularia with two sizes and three morphologies: small (with subtriangular or oblong profile) and large (with sublanceolate profile); and ovicells with more than 18 pseudopores (description based on lectotype, BMNH 1899.5.1.920; see Hayward & Parker [45], Fig 6D–6E). There are no morphological differences between Brazilian specimens studied here and the lectotype of *P*. *bimucronata* (see comparison in Table 3).

*Parasmittina bimucronata* was originally described based on specimens from the Indian Ocean as a variation of *P*. *trispinosa* [44], and the lectotype of that species was selected by Hayward & Parker [47]. Morphological differences can be observed between these two species, including: number of oral spines (1–2 in *P*. *bimucronata* and 2–3 in *P*. *trispinosa*), orientation of the large avicularium (proximolateral in *P*. *bimucronata* and distolateral in *P*. *trispinosa*), and number of ectooecial pseudopores (20–37 in *P*. *bimucronata* and 2–4 in *P*. *trispinosa*) [5, 47]. Hincks [48] referred to specimens from the Pacific Ocean as belonging to *P*. *bimucronata*. However, later, Harmer [36] relocated specimens from Indian and Pacific Oceans studied by Hincks [44, 48] to *P*. *raigii* (Audouin, 1826), originally described from the Red Sea (Gulf of Suez as the type locality). Hayward & Parker [47] reexamined Hincks’ [47, 49] original specimens and stated that none of them belonged to *P*. *raigii*—specimens from the Indian Ocean belong to *P*. *bimucronata* and those from the Pacific were renamed as a new species, *P*. *pectinata* Hayward & Parker, 1994 [47]. Although *P*. *bimucronata*, *P*. *raigii* and *P*. *pectinata* have adventitious avicularia placed laterally to the orifice with similar morphologies, these species can be distinguished mainly by features of the primary orifice, including the distal margin (smooth in *P*. *bimucronata*; beaded in *P*. *raigii* and *P*. *pectinata*), oral spines (1–2 in *P*. *bimucronata* and *P*. *raigii*; 2–3 in *P*. *pectinata*), condyles (thin and smooth in *P*. *bimucronata* and *P*. *raigii*; robust and serrated in *P*. *pectinata*) and lyrula size (narrow in *P*. *bimucronata*; broad *in P*. *raigii* and *P*. *pectinata*). Liu [45] also assigned specimens from China to *P*. *glomerata* due to avicularia with similar morphologies. However, these specimens are here attributed to *P*. *bimucronata*, since it differs from *P*. *glomerata* by having a primary orifice with smooth distal margin (beaded in *P*. *glomerata*) and narrow lyrula (medium-sized in *P*. *glomerata*).

#### Distribution

Indo-Pacific: Myanmar and China [44, 45]; SW Atlantic: Brazil (present study).

#### *Parasmittina dubitata* Hayward, 1980

(Fig 6; Table 10)

*Parasmittina dubitata* Hayward, 1980: 701, figures 2A–B [49].

#### Material examined

Uncatalogued, Tierra del Fuego, Argentina, 90 m.

#### Description

**Fig 7:**
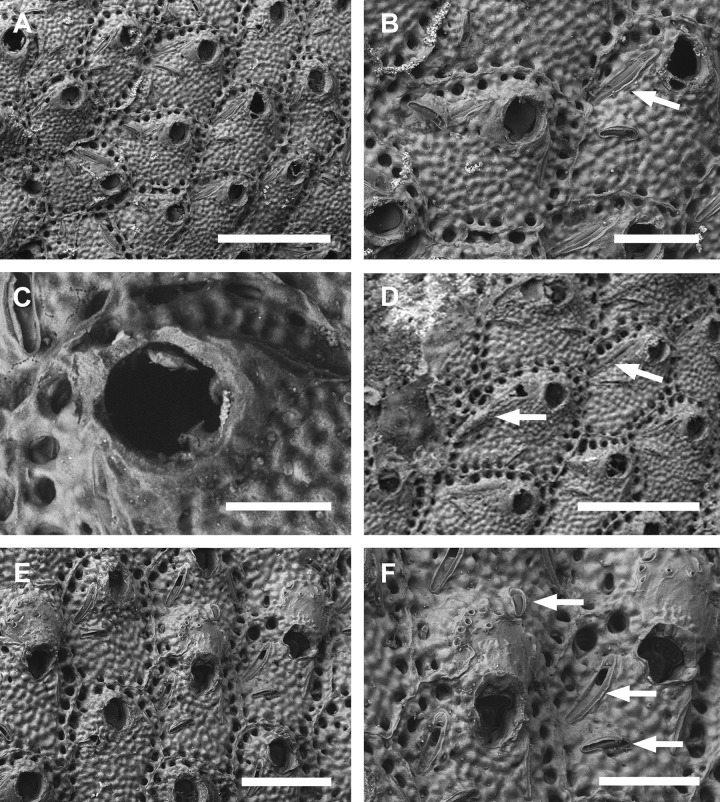
*Parasmittina ligulata* comb. nov. (Ridley, 1881). (A–D) NHMUK 2023.9.22.1, holotype, Espírito Santo, Brazil. Arrows indicating avicularia. (A) group of autozooids showing small avicularia, (B) detail of autozooid showing small avicularia, (C) detail of primary orifice, (D) detail of ovicelled zooids and small avicularia. Scale bars: A = 400 μm; B, D = 200 μm; C = 80 μm.

Colony encrusting. Autozooids (Fig 6A), (0.305–0.403–0.545 mm length; n = 8; SD = 0.096 mm; 0.215–0.267–0.382 mm wide; n = 8; SD = 0.049 mm), subrectangular to polygonal, limited by slightly raised lateral walls, with a single row of 11–16 marginal pores (0.013–0.029–0.059 mm wide; n = 30; SD = 0.010 mm). Frontal wall rugose, with large, rounded tubercles. Primary orifice (Fig 6C), (0.093–0.103–0.120 mm length; n = 7; SD = 0.008mm; 0.086–0.108–0.123 mm wide; n = 7; SD = 0.011mm), subcircular, distal margin smooth, with single oral spine; lyrula large, varying in size, occupy about half width of the orifice (0.034–0.041–0.049 mm wide; n = 7; SD = 0.005 mm), robust, with concave distal margin; a pair of prominent condyles with rounded and coarsely serrated margins. Secondary orifice cormidial, low, forming slightly raised side flaps and a hood distal to the orifice (Fig 6B). Adventitious avicularia variable in shape and size (Fig 6B and 6D), with three morphologies: (1) small (0.059–0.099–0.131 mm length; n = 15; SD = 0.022; 0.017–0.031–0.046 mm wide; n = 15; SD = 0.008 mm), oblong to spatulate avicularia (Fig 6A and 7B) single or paired, laterally at one side of the orifice and oriented proximally or placed near zooidal margins, smooth at its lateral margins, rounded tip, palate occupying one quarter of the rostrum length and elliptical foramen; (2) (0.056–0.066–0.073 mm mm length; n = 4; SD = 0.007; 0.026–0.029–0.033 mm wide; n = 4; SD = 0.003 mm), small, spatulate avicularia (Fig 6A and 6D), placed in zooidal margins, commonly replacing a marginal pore; (3) large (0.241mm in length; n = 1; 0.123 mm in wide; n = 1), subtriangular to sublanceolate avicularia (Fig 6B) (0.425 mm in length; 0.025 mm in wide; n = 1), placed laterally at one side of the orifice, oriented proximally, with acute rostrum, palate broad, foramen elliptical, with smooth margins and crossbar complete. Vicarious avicularia (Fig 6D) with rostrum shorter than autozooids (0.266 mm in length; n = 1; 0.110 mm in wide; n = 1), with the same morphology as the large adventitious avicularium, randomly oriented. Ovicells not observed.

#### Remarks

*Parasmittina dubitata* was first described based on specimens from Burdwood Bank, Argentina, growing on organic carbonates [49]. Here, we provide characterization of a single specimen from Tierra del Fuego, Argentina, kindly sent by Juan Lopez Gappa (pers. comm. 2021), that exhibts the diagnostic characters of the original description of *P*. *dubitata*. This species is readily distinguished from other congeners by the primary orifice morphology, including a lyrula with a concave distal margin and rounded condyles, unlike the straight lyrula and triangular to hooked condyles of most *Parasmittina* species.

#### Distribution

SW Atlantic: Argentina (Burdwood Bank and Tierra del Fuego) [49] present study).

### *Parasmittina ligulata* (Ridley, 1881) comb. nov.

(Fig 7, Table 10)

*Smittia trispinosa* var. *ligulata* Ridley, 1881: 53, plate 6, figure 9 [50].

#### Material examined

*Holotype*: NHMUK 2023.9.22.1, *Smittia trispinosa* var. *ligulata*, S.O. Ridley det., Alert Circumnavigation 1878–1882, HMS Alert, Victoria Bank, Espírito Santo, Brazil, 60 m.

#### Redescription

Colony encrusting. Autozooids (0.324–0.458–0.603 mm length; n = 15; SD = 0.073 mm; 0.209–0.324–0.462 mm wide; n = 15; SD = 0.072 mm), (Fig 7A) polygonal, almost rhombic, limited by slightly raised lateral walls, surrounded by a single row of 19–27 marginal pores (0.010–0.019–0.032 mm in diameter; n = 30; SD = 0.004 mm). Frontal wall with rugose and nodular calcification (Fig 7A). Primary orifice (0.063–0.093–0.112 mm length; n = 4; SD = 0.023 mm; 0.074–0.087–0.096 mm wide; n = 4; SD = 0.009 mm) (Fig 7C) elliptical, distal margin smooth, oral spines not observed, lyrula narrow (0.015–0.021–0027 mm wide; n = 4; SD = 0.005 mm); a pair of robust and down-curved hooked condyles with coarsely serrated margins (Fig 7B). Secondary orifice (Fig 7B and 7F) pear-shaped, forming lateral flaps, resulting in a shallow U-shaped pseudosinus (Fig 7E and 7F). Adventitious avicularia variable in shapes and sizes (Figs 7B, 7D and 7F), with three morphologies: (1) small, narrow, and elongated avicularia (Fig 7B, 7D and 7F) (0.108–0.163–0.210 mm in length; n = 10; SD = 0.034 mm; 0.015–0.022–0.032 mm in wide; n = 10; SD = 0.005 mm), present at one or both side of the orifice, unequal in size if paired, placed below the secondary orifice, proximally oriented, rostrum narrow and slightly curved, smooth to slightly corrugated at its lateral margins, rounded tip, palate occupying about half of the rostrum length and oblong foramen; (2) small, narrow and elliptic avicularia (Fig 7B and 7F) (0.060–0.083–0.116 mm in length; n = 10; SD = 0.014 mm; 0.013–0.017–0.025 mm in wide; n = 10; SD = 0.003 mm), placed o zooidal margins and/or o the frontal wall, with smooth lateral margins, rounded tip, palate narrow and elliptical foramen; (3) large, sub-lanceolate avicularia (Fig 7D), placed laterally at one side of the orifice, oriented proximally, rostrum slightly curved with rounded distal edge, palate narrow and calcified, occupying more than half of avicularium chamber, foramen elliptical, crossbar complete, without columella, Ovicells (0.171–0.185–0.211mm length; n = 5; SD = 0.017 mm; 0.163–0.188–0.212 mm wide; n = 5; SD = 0.022 mm) globose, densely surrounded by adjacent frontal wall, ectooecium with 6–10 medium–sized pores (Fig 7F).

#### Remarks

*Parasmittina ligulata* comb. nov. belongs to the complex *P*. *spathulata*–*P*. *areolata*, that comprise taxa from the Atlantic and Pacific Oceans with single or paired adventitious latero-oral avicularia with narrow and elongated profile (e.g., [3, 7, 21]).

*Parasmittina ligulata* comb. nov. was originally described based on specimens from southeastern Brazil, but later synonymized under *P*. *spathulata* by Vieira *et al*. [25], who also include in the same synonym list Brazilian specimen attributed to *Smittina trispinosa spathulata* (USNM 8558). As stated above, the specimen studied by Canu & Bassler [40] belongs to *P*. *abrolhosensis*. Also, the analysis of the syntype showed that *P*. *ligulata* comb. nov. can be readily differentiated from *P*. *spathulata* and *P*. *abrolhosensis* by showing large, avicularia elongate to sublanceolate avicularia (spatulate in the other species). Other differences between *P*. *ligulata* comb. nov. and *P*. *spathulata* is lyrula size (occupying one-quarter of the orifice in *P*. *ligulata* comb. nov. and half of the orifice in *P*. *spathulata*), and between *P*. *ligulata* comb. nov. and *P*. *abrolhosensis* is the occurrence of small latero-oral subtriangular avicularia in *P*. *abrolhosensis* (absent in *P*. *ligulata* comb. nov.).

Among all congeners, *P*. *ligulata* comb. nov. most resembles *Parasmittina longirostrata* Liu in Liu, Yin & Ma, 2001 [45] mainly because of small proximolateral, elongate and large sublanceolate avicularia. However, these species are distinguished by lyrula size (occupying about one-quarter of the orifice in *P*. *ligulata* comb. nov. and half of the orifice in *P*. *longirostrata*) and small oblong avicularia (absent in *P*. *ligulata* comb. nov. and very common in *P*. *longirostrata*).

#### Distribution

SW Atlantic: Brazil [50].

### *Parasmitina longirostrata* Liu in Liu, Yin & Ma, 2001

**Fig 8:**
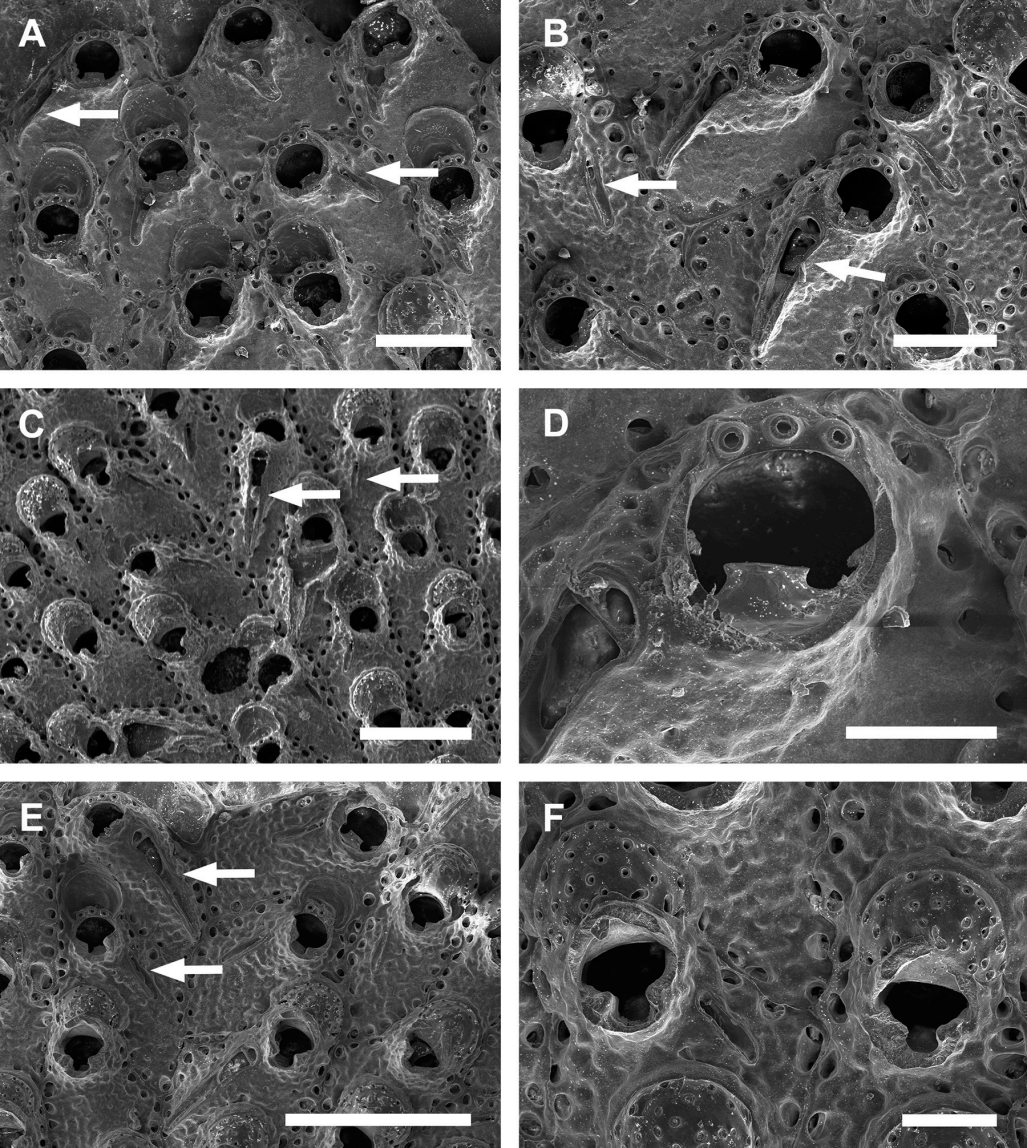
*P*. *longirostrata* Liu in Liu, Yin & Ma, 2001. (A–F) UFBA 5444, Bahia, Brazil. Arrows indicating avicularia. (A) general view of a colony, (B) autozooids at the growing edge of the colony, (C) autozooids with large and small latero-oral avicularia, (D) detail of primary orifice, (E) detail of zooids with large and small avicularia, (F) group of ovicelled zooids. Scale bars: A, B, C, E = 200 μm; F, D = 100 μm.

(Fig 8; Tables 4, 10)

**Table 4:** Morphometric data of *P*. *longirostrata* and *P*. *serruloides*. Values given in millimeters and represented as minimum–median–maximum (number of zooidal measures). Avicularia abbreviations: Av1 = small elongate; Av2 = large sublanceolate.

Character	Taxa / Reference		
	*P*. *longirostrata* / Liu *et al*. (2001)	*P*. *longirostrata* / UFBA 5444 (present study)	*P*. *serruloides* / Harmelin *et al*. (2009)
**ZL**	0.480–0.600–0.744 (10)	0.316–0.449–0.634 (15)	0.270–0.375–0.460 (29)
**ZW**	0.314–0.342–0.358 (10)	0.205–0.294–0.410 (15)	0.180–0.242–0.300 (29)
**OL**	0.098–0.100–0.106 (10)	0.073–0.084–0.096 (10)	-
**OW**	-	0.084–0.092–0.104 (10)	-
**Av1L**	0.072–0.120–0.144 (10)	0.074–0.128–0.164 (7)	0.90–0.115–0.145 (29)
**Av1W**	0.028–0.042–0.056 (10)	0.012–0.018–0.025 (7)	-
**Av2L**	0.330–0.369–0.414 (10)	0.257–0.298–0.327 (8)	0.195–241.5–0.330 (26)
**Av2W**	0.174–0.218–0.258 (10)	0.031–0.039–0.048 (8)	-
**OvL**	0.142–0.158–0.172 (10)	0.136–0.153–0.169 (15)	0.125–0.149.2–0.160 (23)
**OvW**	0.186–0.204–0.214 (10)	0.167–0.184–0.202 (15)	0.60–0.184–0.205 (23)

*Parasmittina longirostrata* Liu *et al*. 2001: 800, plate 53, figures 4–6 [45].

?*Parasmittina serruloides* Harmelin *et al*. 2009: 174, figure 6A–D [9].

#### Material examined

UFPE 816–821, UFBA 5030, 5097, 5198, 5213, 5248, 5297, 5343, 5349, 5354, 5360, 5365–5366, 5370–5372, 5374, 5389, 5396, 5400, 5407, 5409, 5414, 5416, 5422, 5427, 5430, 5431, 5436, 5438, 5444, 5453, 5465, Baia de Todos os Santos, Salvador, Bahia, Brazil, 3–5 m, coll. 2012.

#### Description

Colony encrusting (Fig 8A). Autozooids (Fig 8B) subrectangular to polygonal, limited by slightly raised lateral walls, surrounded by a single row of 12–18 small marginal pores (0.004–0.013–0.025 mm in diameter; n = 30; SD = 0.004 mm). Frontal wall rugose and nodular. Primary orifice elliptical (Fig 8C), wider than long, distal margin smooth, with 3–4 oral spines, lyrula median-sized (0.025–0.030–0.036 mm wide; n = 15; SD = 0.003 mm), a pair of robust, hooked, condyles with serrated margins (up to seven teeth). Secondary orifice (Fig 8D and 8F) moderate to well-developed, pear-shaped, forming 2–4 lateral flaps commonly resulting in a shallow U-shaped pseudosinus in autozooids, only partly obscuring the lyrula, with a hood-shaped distal calcification in ovicelled zooids. Adventitious avicularia variable in shapes and sizes (Fig 8B–8D), with two morphologies: (1) small, narrow and elongate avicularia (Fig 8B and 8D) present at one side below to the secondary orifice, proximally oriented, rostrum narrow and slightly curved, slightly corrugated at its lateral margins, rounded tip, palate occupying about half of the rostrum length, oblong foramen and crossbar complete; (2) large, sub-lanceolate avicularia (Fig 8C and 8E), placed laterally at one side of the orifice, oriented proximally, rostrum slightly curved with coarsely serrated margins and rounded distal edge, palate narrow, occupying about half the rostrum length, foramen subtriangular with 2–3 pseudopores adjacent to the avicularia. Ovicells globose, initially prominent (Fig 8D, and 8F) and densely surrounded by adjacent frontal wall (Fig 8F) in older zooids; ectooecium with 18–24 medium-sized pseudopores (0.003–0.006–0.009 in diameter; n = 30; SD = 0.001 mm), regularly distributed through the ectooecial surface.

#### Remarks

*Parasmittina longirostrata* is characterized by the combination of a primary orifice with smooth distal margin, with 3–4 oral spines, lyrula median-sized, secondary orifice forming a U-shaped pseudosinus, and two types of proximolateral avicularia: small, elongate and thin, and large, sub-lanceolate with crenulated margins [45].

Liu *et al*. [45] considered that *P*. *serrula* Soule & Soule, 1973 [2] was the most similar congener to *P*. *longirostrata* probably because of having two types of avicularia with the smaller and proximolateral being thin and narrow. However, several differences are observed between these species, including the primary orifice (with smooth distal margin and lyrula occupying half of the orificial width in *P*. *longirostrata* and with distal denticles and lyrula occupying one-quarter of the orificial width in *P*. *serrula*), placement of the smaller avicularia (typically straightly placed proximolateral to the orifice in *P*. *longirostrata* and transversally below the orifice in *P*. *serrula*) and profile of the large avicularium (sublanceolate in *P*. *longirostrata* and lanceolate with lobate edge at half distal part in *P*. *serrula*). A congener very similar to *P*. *longirostrata*, however, is *P*. *serruloides* Harmelin et al. 2009 [9], described based on specimens from the Mediterranean. These species are only distinguished by the profile of the condyles (robust and with 4–7 teeth in *P*. *longirostrata*, but thin and with 2–5 teeth in *P*. *serruloides*). However, the profile of the condyles can be variable within a colony, as already seen in other *Parasmittina* (e.g., *P*. *onychorrhyncha* Ryland & Hayward, 1992 and *P*. *aleutensis* Soule & Soule, 2002). Thus, despite the type locality of *P*. *longirostrata* being in the North Pacific. The morphology and morphometry of the specimens from Brazil resemble those of specimens of *P*. *longirostrata* from China [45], but the autozooids of *P*. *serruloides* appear to be smaller zooids when compared with *P*. *longirostrata* (Table 4). However, further studies are needed with specimens from the Mediterranean, to determine if there are intraspecific differences that justify the synonymization of *P*. *serruloides* with *P*. *longirostrata*

#### Distribution

Northeastern Pacific: China [45]; SW Atlantic: Brazil (present study). Specimens from Lebanon [9] need to be compared to ascertain the synonym with *P*. *longisrostrata*.

#### *Parasmittina pinctatae* Liu in Liu, Yin & Ma, 2001

**Fig 9:**
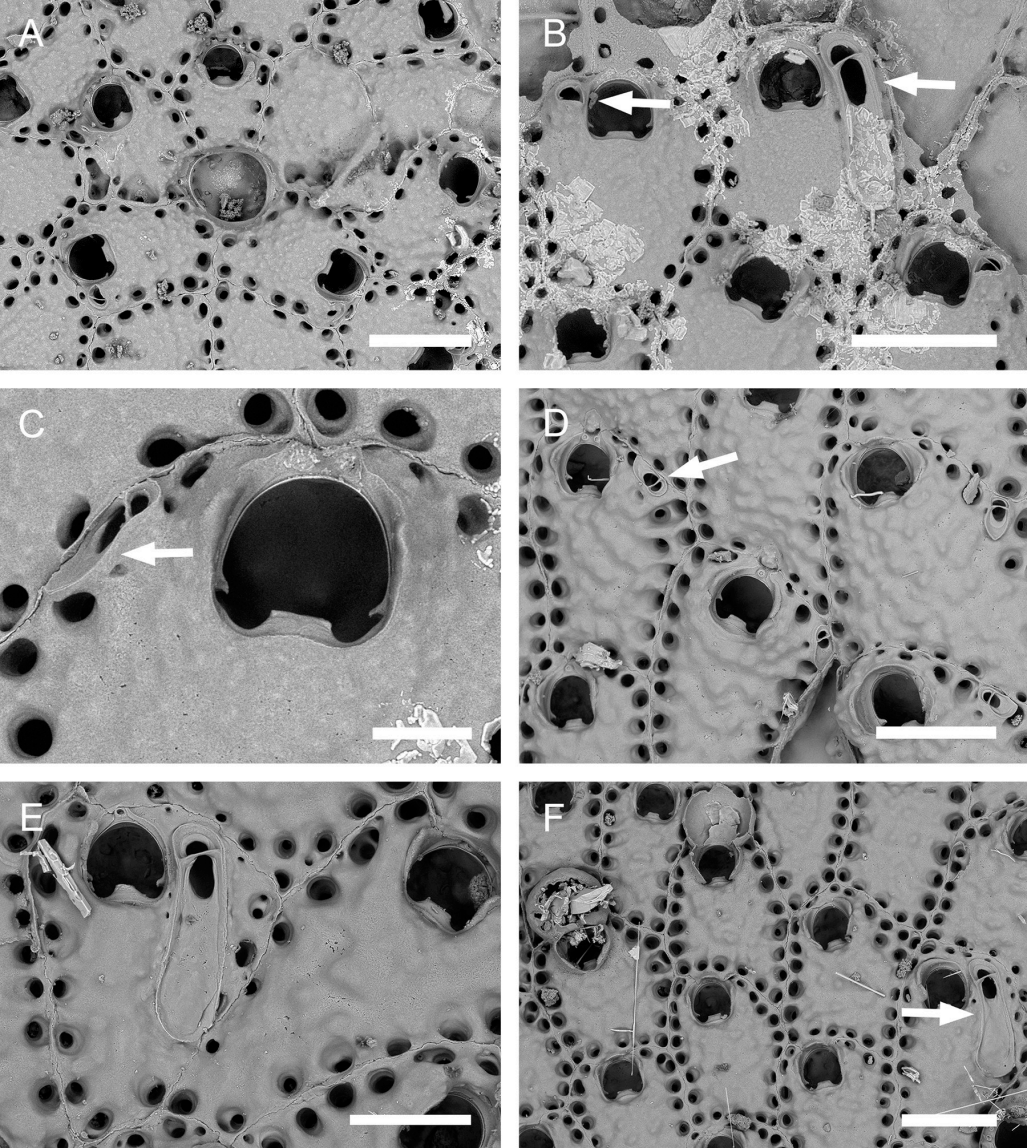
*Parasmittina pinctatae* Liu in Liu, Yin & Ma, 2001. (A–C) UFBA 5447, (D–F) UFBA 5453, Bahia, Brazil. Arrows indicating avicularia. (A) ancestrula and first zooids of the colony, (B) young autozooids showing small and large avicularia, (C) detail of primary orifice and small avicularium, (D) group of autozooids showing small avicularia, (E) detail of autozooids and large avicularium, (F) autozooids and ovicelled zooids. Scale bars: A, B, D–F = 250 μm; C = 50 μm.

**Fig 10:**
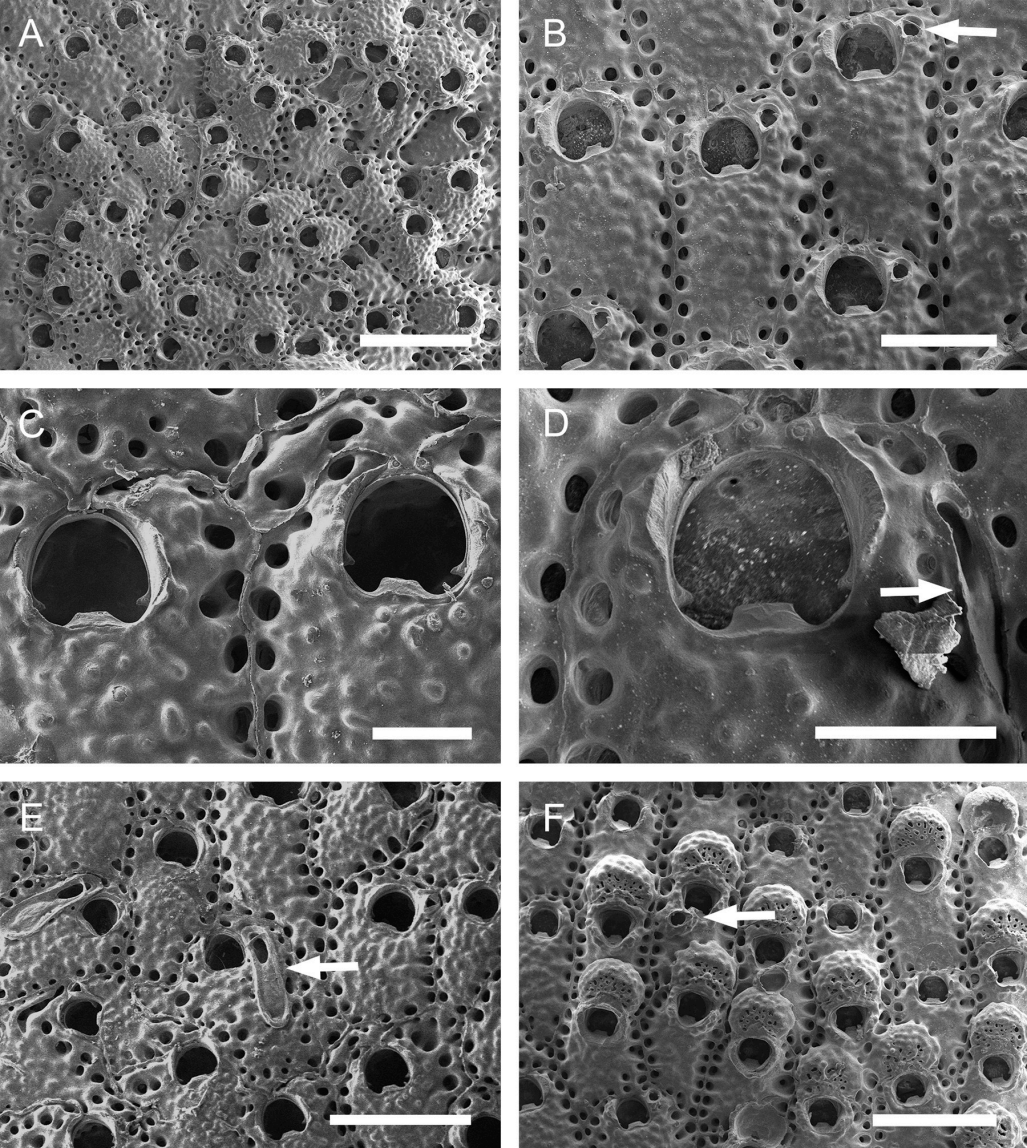
*Parasmittina pinctatae* Liu in Liu, Yin & Ma, 2001. (A–D, F) UFBA 5204, (C, E) UFBA 5466, Bahia, Brazil. Arrows indicating avicularia. (A) general view of a colony, (B) autozooids at the growing edge of the colony showing small avicularia, (C) detail of primary orifices, (D) detail of primary orifice with small avicularia, (E) group of autozooids with small and large avicularia, (F) group of ovicelled zooids with secondary calcification. Scale bars: A, F = 500 μm; B, D = 200 μm; C = 100 μm; E = 400 μm.

(Figs 9, 10; Tables 5, 10)

**Table 5:** Morphometric data of *P*. *pinctatae*, *P*. *parsevalii* and *P*. *egyptiaca*. Values given in millimeters and represented as minimum–median–maximum (number of zooidal measures), (*) = In Liu *et al*. (2001) there is an error in the median value, which is why it was adopted this way Avicularia abbreviations: Av1 = small subtriangular; Av2 = small elongate; Av3 = large spatulate.

Character	Taxa / Reference				
	*P*. *pinctatae /* Liu *et al*. (2001)	*P*. *pinctatae /* UFBA 5204 (present study)	*P*. *parsevalii /* Liu *et al*. (2001)	*P*. *egyptiaca /* Harmelin *et al*. (2009)	*P*. *egyptiaca /* Baradari *et al*. (2019)
**ZL**	0.483–0.522–0.655 (10)	0.380–0.467–0.672 (15)	0.366–0.498–0.670 (10)	0.365–0.492–0.730 (33)	0.270–0.397–0.531 (13)
**ZW**	0.277–0.320–0.350 (10)	0.223–0.270–0.351 (15)	0.280–0.302–0.340 (10)	0.265–0.338–0.605 (33)	0.230–0.266–0.320 (13)
**OL**	0.112–0.122–0.135 (10)	0.091–0.104–0.114 (15)	0.098–0.106–0.110 (10)	0.085–0.107–0.130 (28)	-
**OW**	0.088–0.098–0.102 (10)	0.088–0.099–0.114 (15)	0.084–0.092–0.098 (10)	0.105–0.113–0.135 (28)	-
**Av1L**	0.064–0.084–0.098 (10)	0.035–0.053–0.090 (10)	*0.084–0.090 (10)	-	-
**Av1W**	0.036–0.040–0.064 (10)	0.023–0.032–0.044 (3)	0.050–0.062–0.072 (10)	-	-
**Av2L**	0.088–0.102–0.110 (10)	0.137 (1)	0.072–0.080–0.086 (10)	-	-
**Av2W**	0.038–0.048–0.056 (10)	0.015 (1)	0.048–0.054–0.072 (10)	-	-
**Av3L**	0.292–0.304–0.316 (10)	0.290–0.319–0.337 (3)	0.240–0.254–0.268 (10)	0.315–0.371–0.435 (10)	-
**Av3W**	0.074–0.080–0.088 (10)	0.057–0.061–0.066 (3)	0.098–0.098–0.114 (10)	0.085–0.108–0.120 (10)	-
**OvL**	0.180–0.200–0.216 (10)	0.129–0.173–0.192 (15)	0.184–0.198–0.220 (10)	0.195–0.205–0.230 (16)	–
**OvW**	0.208–0.240–0.245 (10)	0.163–0.221–0.243 (15)	0.224–0.236–0.256 (10)	0.205–0.236–0.265 (16)	–

*Parasmittina pinctatae* Liu in Liu, Yin & Ma, 2001: 805, plate 58, figures 3–6 [45].

*Smittina trispinosa*: Luederwaldt 1929: 65 [49]. Non *Discopora trispinosa* Johnston, 1838: 280 [51].

*Smittina trispinosa* var. *nitida*: Marcus 1937: 104, plate 21, figure 56 [23]. Non *Discopora nitida* Verril, 1875: 415 [51].

*Parasmittina betamorphaea*: Vieira *et al*. 2008: 27 [25]. Non *Parasmittina betamorphaea* Winston, 2005: 58 [7].

*Parasmittina parsevalii*: Liu *et al*. 2001: 621, plate 54, figures 5–7 [46]. Non *Cellepora parsevalii* Audouin, 1826: 238 [5].

*Parasmittina egyptiaca*: Harmelin *et al*. 2009: 166, fig 2A–2E [9]; Baradari *et al*. 2019: 474, figures 54–57 [18]. Non *Smittia egyptiaca* Waters, 1909: 157 [52].

#### Material examined

UFBA 301, 644, 1112, 5466, Todos os Santos, Bahia, Brazil, 37 m, coll. 17/04/1997; UFBA 5317, Praia do Francês, Marechal Deodoro, Alagoas, Brazil, coll. 03/02/2003; UFPE 2787, Porto do Cabedelo, Cabedelo, Paraíba, Brazil, intertidal, coll. 27/09/2009; UFPE 2582–2583, Praia Araçá, São Sebastião, São Paulo, Brazil, coll. 07/07/2009; USNM Uncatalogued, H. Luederwaldt det. 1926, Ilha de São Sebastião, São Paulo, Brazil; UFPE 822–830, UFBA 5011, 5022, 5025, 5031, 5039, 5050, 5055, 5059, 5066, 5070, 5075, 5080, 5085, 5089, 5094, 5100, 5104, 5110, 5116, 5127, 5130, 5136, 5144, 5151, 5157, 5168, 5177, 5193, 5195, 5204, 5216, 5223, 5230, 5236, 5241, 5245, 5252, 5259, 5260, 5265, 5270, 5278–5279, 5287, 5291, 5298, 5345, 5351, 5358, 5363, 5373, 5384, 5386, 5397, 5417, 5424, 5428, 5451, 5454, 5457, 5461, 5466, 5497, 5503, 5506–5507, Baia de Todos os Santos, Salvador, Bahia, Brazil, 3–5 m, coll. 2012; MZUSP 921, *Smittina trispinosa* var. *nitida*, det. Marcus, 1937, no locality [supposedly Santos, São Paulo, Brazil]; MZUSP 1160, Iate Clube Ilhabela, Ilhabela, São Paulo, Brazil, 2 m, coll. 05/03/2010; MZUSP 2430.3, CE59, Porto do Mucuripe, Fortaleza, Ceará, Brazil, coll. 27/08/2009; MZUSP 2952, CE65, Porto do Mucuripe, Fortaleza, Ceará, Brazil, 3°32’S, 38°48’W, coll. 27/08/2009; MZUSP 2420, 2431.1, CE67, Porto do Mucuripe, Fortaleza, Ceará, Brazil, coll. 27/08/2009; MZUSP 2953, CE69, Porto do Mucuripe, Fortaleza, Ceará, Brazil, coll. 27/08/2009; MZUSP 2515, Ubatubinha, Ilha Grande, Angra dos Reis, Rio de Janeiro, Brazil,… . coll, 15/10/2012; MZUSP 1015, *Smittina trispinosa* var. *nitida*, det. Marcus, 1937, no locality [supposedly Santos, São Paulo, Brazil]; USNM Uncatalogued, *Smittina trispinosa*, det. H. Luederwaldt, São Sebastião, São Paulo, Brazil, coll. Jul 1925.

#### Description

Colony encrusting, uni to multilaminar (Fig 9A). Ancestrula (Fig 9A) tatiform, without marginal cryptocyst, 1 marginal spine; 3 orificial spines in first daughter zooid. Autozooids (Figs 9B, 10B) subquadrangular to polygonal, limited by slightly raised lateral walls, surrounded by a single row of 16–22 marginal pores (0.011–0.022–0.032 mm in diameter; n = 30; SD = 0.004 mm). Frontal wall rugose and nodular. Primary orifice (Figs 9C and 10C) subcircular, as long as wide, smooth distally, 1–2 oral spines, lyrula relatively wide but low, occupying about half of the orifice length (0.025–0.038–0.047 mm wide; n = 15; SD = 0.006 mm), a pair of almost straight and robust condyles with fine serrated margins. Secondary orifice low, forming short lateral flaps (Figs 9D, 10C), larger in ovicelled zooids (Figs 9F, 10F). Adventitious avicularia variable in shape and size (Fig 9B–9D), with three morphologies: (1) small subtriangular avicularia (Figs 9B and 10B, 10E), placed distolaterally to the orifice in younger zooids and randomly scattered on the frontal wall (i.e., around the orifice, and on the ovicell) in older zooids (Fig 10F); (2) small, profile elongate, sublanceolate (Figs 9C, 9E and 10C), to spatulate (Fig 10C), narrow and with pointed rostrum, placed o zooidal margins, more common in older zooids; (3) large, profile spatulate (Figs 9B, 9E and 10E), placed distolaterally to the orifice, oriented proximolaterally, palate narrow, occupying about three-quarters of the rostrum length, with smooth margins and crossbar complete; 3–5 pseudopores adjacet to the avicularia, foramen elliptical. Ovicells globose (Figs 9F and 10F), densely surrounded by adjacent frontal wall (Fig 10F); ectooecium with more than 20 small to medium-sized pseudopores (0.002–0.007–0.011 in diameter; n = 30; SD = 0.002 mm) distributed through the entire ovicell surface.

#### Remarks

Although *P*. *pinctatae* occurs in Brazil since at least 1929, due to misleading identifications [23, 26, 53], we present the first formal record of this species to the Western Atlantic. In Brazil, this species was previously attributed to *P*. *trispinosa* [32, 53], and *P*. *nitida* (Verrill, 1875) but Vieira *et al*. [25] misassigned Marcus’s *P*. *nitida* to *P*. *betamorphaea*. Here we examined specimens studied by several authors (Luederwaldt [53], USNM Uncatalogued; Marcus [23], MZUSP 921; Almeida *et al*. [26], UFBA 301 and UFBA 644), and all share with *P*. *pinctatae* the subcircular primary orifice, secondary orifice with lateral flaps, short lyrula, nodular frontal calcification and three types of avicularia. Additionally, specimens recently assigned to *P*. *egyptiaca* by Orr *et al*. [54] from Brazil (Bahia) and Spain belong to *P. pinctatae (Table S2 of supplementary material from [54])*.

*Parasmittina pinctatae* differs from *P*. *trispinosa* in the profile of the primary orifice (as long as wide in *P*. *pinctatae* and longer than wide in *P*. *trispinosa*), number of oral spines (1–2 in *Parasmittina pinctatae* and 2–3 in *P*. *trispinosa*), profile of avicularia (the proximolateral is subtriangular in *P*. *pinctatae* and oblong in *P*. *trispinosa*; the large is spatulate in *P*. *pinctatae* and subtriangular in *P*. *trispinosa*), and number of ovicell pseudopores (more than 20 in *P*. *pinctatae* and 2–4 in *P*. *trispinosa*) [5]. Differences between *P*. *pinctatae* and *P*. *nitida* include the profile of the primary orifice (subcircular in *P*. *pinctatae* and rounded in *P*. *nitida*), lyrula (median in *P*. *pinctatae* and narrow in *P*. *nitida*), types of avicularia (three types in *P*. *pinctatae*: proximolateral, marginal and large; two types in *P*. *nitida*: proximolateral and marginal) and orientation of the proximolateral avicularia (distolateral in *P*. *pinctatae* and proximolateral in *P*. *nitida*) [55]. Finally, *P*. *pinctatae* is distinguished from *P*. *betamorphea* in having a narrower lyrula (the lyrula of *P*. *betamorphaea* is twice the length of that from of *P*. *pinctatae*) and condyles thin and almost straight (robust and curved in *P*. *betamorphaea*) [7].

The taxonomic history of *P*. *pinctatae* needs to be explained. Liu *et al*. [45] attributed other specimens from China to *P*. *parsevalii* (Audouin, 1826) due to these having avicularia placed at the frontal wall and laterally to the orifice (not associated with the peristome like in *P*. *pinctatae*) and differences in ooecia surface. However, *P*. *parsevalii sensu* Liu *et al*. [45] differs from *P*. *parsevalii* (acc. Hayward & Parker [47]) by having a primary orifice with smooth distal margin (beaded in *P*. *parsevalii*), relatively wide and low lyrula (narrow and high in *P*. *parsevalii*), almost straight and thin condyles (robust and hooked in *P*. *parsevalii*) and large spatulate avicularia (subtriangular in *P*. *parsevalii*). Thus, here we reassign the Chinese specimens of *P*. *parsevalii* to *P*. *pinctatae*.

**Fig 11:**
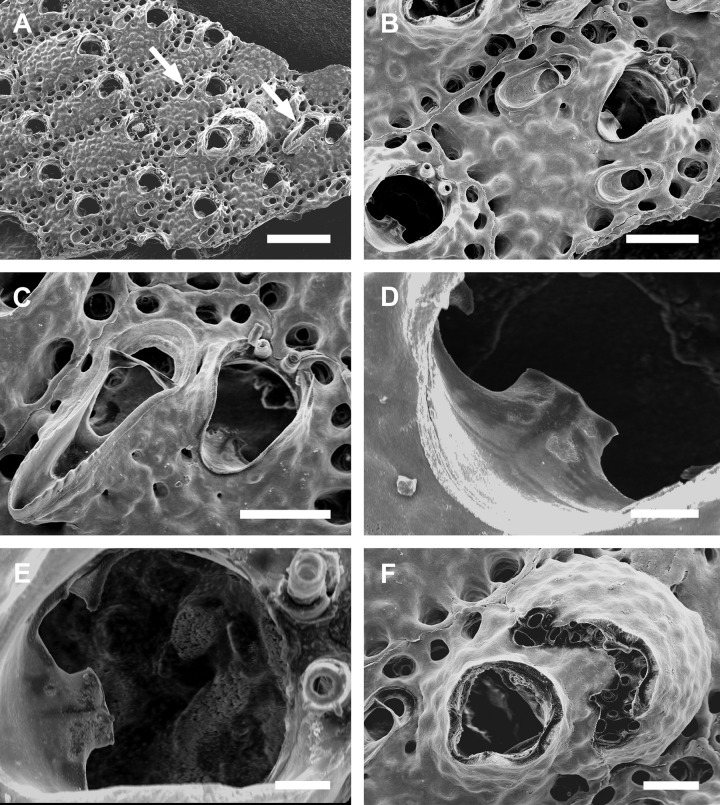
*Parasmittina egyptiaca* (Waters, 1909). (A–F) MFN 1143, type. Arrows indicating avicularia. (A) colony fragment showing small and large avicularia, (B) detail of autozooid showing oral spines and small avicularia, (C) detail of autozooid showing large avicularia, (D) detail of primary orifice showing condyles and lyrula, (E) detail of primary orifice showing oral spines and slightly denticulate distal margin, (F) detail of ovicell. Scale bars: A = 300 μm; B, C, F = 100 μm; D, E = 20 μm.

Some records of *P*. *egyptiaca* made by Harmelin *et al*. [9, 56] and Baradari *et al*. [18] also belong to *P*. *pinctatae*. The type specimen of *P*. *egyptiaca* (MFN Uncatalogued; Fig 11) is distinct from specimens characterized by these authors by having a raised secondary orifice, large avicularia with subtriangular profile and serrated margins and latero-oral avicularia oriented proximally. Thus, a review of specimens previously attributed to both *P*. *parsevalii* and *P*. *egyptiaca* and morphologically related is strongly recommended.

#### Distribution

Central Pacific: China [45]; Red Sea [52]; Mediterranean: Lebanon [9, 56]; Persian Gulf: Iran [18]; SW Atlantic: Brazil [26, 23, 53] present study).

### *Parasmittina serrula* Soule & Soule, 1973

**Fig 12:**
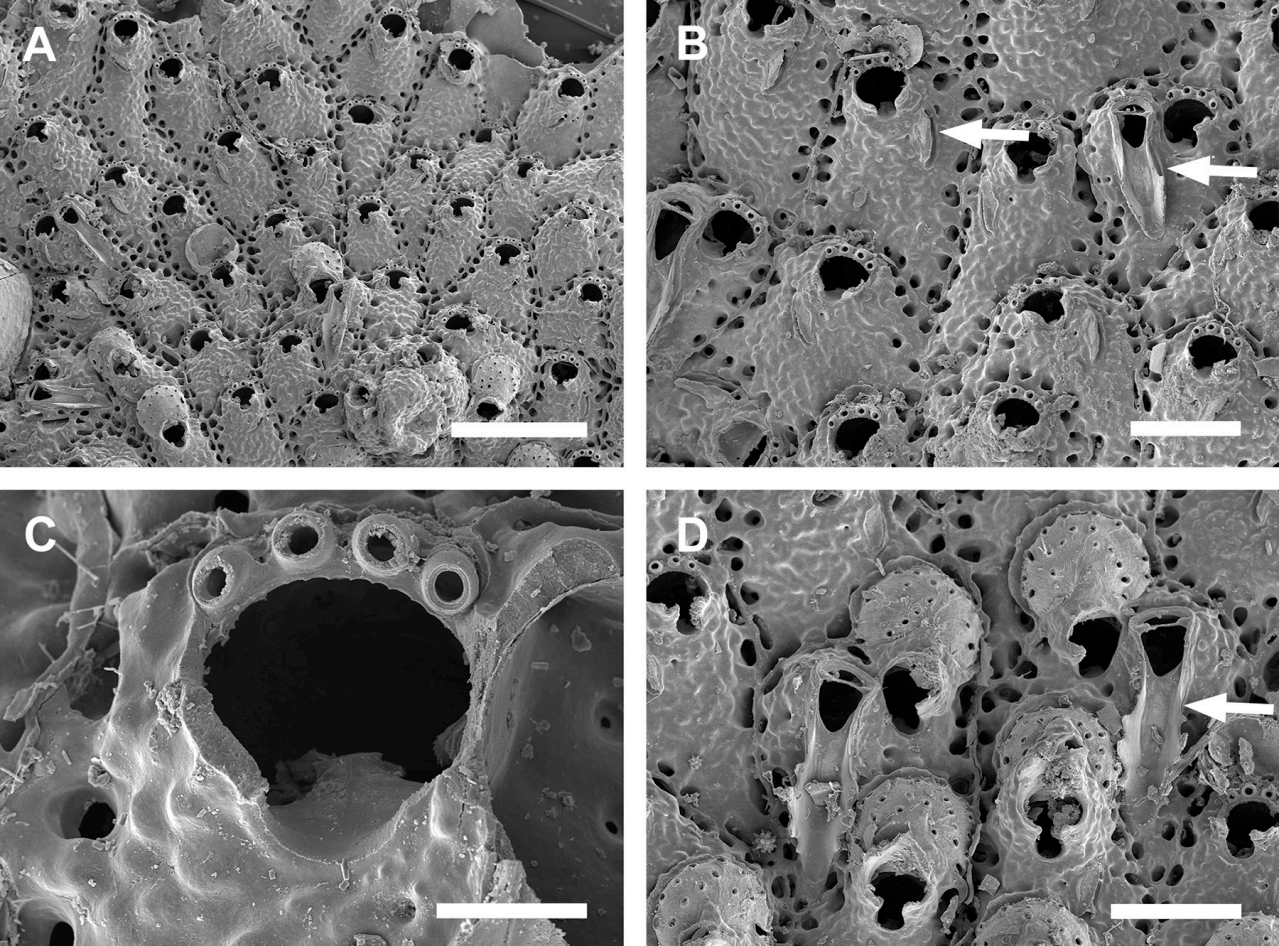
*Parasmittina serrula* Soule & Soule, 1973. (A–D) UFBA 2850, Bahia Brazil. Arrows indicating avicularia. (A) general view of a colony, (B) autozooids at the growing edge of the colony showing small and large avicularia, (C) detail of primary orifice, (D) group of ovicelled zooids showing large avicularia. Scale bars: A = 500 μm; B, D = 200 μm; C = 50 μm.

(Fig 12; Tables 6, 7, 10)

**Table 6:** Morphometric data of *P*. *serrula* and *P*. *luteoserrula*. Values given in millimeters and represented as minimum–median–maximum (number of zooidal measures). Avicularia abbreviations: Av1 = small elongate; Av2 = large spatulate.

Character	Taxa / Reference		
	*P*. *serrula /* Soule & Soule (1973)	*P*. *serrula* / UFBA 2850 (present study)	*P*. *luteoserrula* / Winston & Jackson (2021)
**ZL**	0.320–0.368–0.400 (10)	0.309–0.440–0.637 (15)	0.486–0.593–0.738 (18)
**ZW**	0.260–0.284–0.310 (10)	0.190–0.257–0.310 (15)	0.288–0.363–0.450 (18)
**OL**	0.080–0.084–0.090 (10)	0.065–0.084–0.120 (10)	0.072–0.085–0.108 (18)
**OW**	0.060–0.066–0.075 (10)	0.074–0.087 0.102 (10)	0.090–0.105–0.126 (18)
**Av1L**	0.075–0.079–0.080 (6)	0.083–0.099–0.127 (15)	**-**
**Av1W**	0.030–0.031–0.035 (6)	0.004–0.015–0.026 (15)	**-**
**Av2L**	0.240–0.252–0.260 (5)	0.255–0.309–0.359 (6)	0.252–0.323–0.378 (18)
**Av2W**	0.060–0.063–0.065 (5)	0.051–0.057–0.066 (6)	0.072–0.091–0.126 (18)
**OvL**	0.220 (1)	0.118–0.152–0.170 (15)	0.162–0.185–0.216 (18)
**OvW**	0.240 (1)	0.180–0.195–0.220 (15)	0.180–0.199–0.360 (18)

**Table 7:** Morphological variations in specimens from different localities assigned to *P*. *serrula* and *P*. *luteoserrula*. Unknown states are assigned as (?).

Taxa / Localities	Reference	Oral spines	Condyles profile	Lyrula and orifice width ratio
***P*. *serrula***				
Hawaii (EUA), Central Pacific	Soule & Soule (1973)	3–6	strong	0.25
Hawaii (EUA), Central Pacific	Gordon (1984)	4	?	0.25
Heron Island (Australia), Coral Sea	Ryland & Hayward (1992)	2–4	strong	0.50
Philippines, Southwest Pacific	Gordon & D’Hondt (1997)	3	?	?
Vanuatu, Southwest Pacific	Tilbrook *et al*. (2001)	2–4	strong	0.50
Vanuatu, Southwest Pacific	Tilbrook *et al*. (2006)	3–4	thin	0.25
Hawaii (EUA), Central Pacific	Dick *et al*. (2006)	2–4	strong	0.33
Okinawa (Japan), Central Pacific	Dick & Grischenko (2017)	3–4	strong	0.50
Brazil, Southwestern Atlantic	Present study	3–5	strong	0.25
***P*. *luteoserrula***				
Kingston (Jamaica), Caribbeann	Winston & Jackson (2021)	3–4	strong	0.25–0.33

*Parasmittina serrula* Soule & Soule, 1973: 386, fig 3D–F [2].

? *Parasmittina luteoserrula* Winston & Jackson, 2021: 141 [13].

#### Material examined

UFBA 2850, Guarajuba, Camaçari, Bahia, Brazil, 25–27 m, coll. 1997; UFBA 2859, Recife dos Cascos, Baia de Todos os Santos, Bahia, Brazil, 13–21 m, coll. 2016–2017; MZUSP 2944, CE24, Canal das Arabaianas, Ceará, Brazil.

#### Description

Colony encrusting, uni to multilaminar (Fig 12A). Autozooids (Fig 12B) rhombic-polygonal, limited by slightly raised lateral walls, surrounded by a single row of 14–22 marginal pores (0.009–0.019–0.036 mm wide; n = 30; SD = 0.005 mm). Frontal wall rugose and nodular. Primary orifice (Fig 12C) elliptical, wider than long, beaded distal margin with several well-spaced minute denticles, 3–5 oral spines, lyrula narrow, occupying about half of the orifice length (0.020–0.023–0.028 mm wide; n = 10; SD = 0.002 mm), a pair of hooked condyles with coarsely serrated margins. Secondary orifice pear-shaped, forming 2 proximolateral flaps and resulting in a pseudosinus (Fig 12B and 12D), larger in ovicelled zooids (Fig 12D). Adventitious avicularia variable in shapes and sizes (Fig 12A, 12B and 12D), with two morphologies: (1) initially small, narrow and elliptical (Fig 12A), becoming elongate with development (Fig 12B), present at one or both sides below the secondary orifice, proximally oriented, rostrum narrow and slightly curved, slightly corrugated at its lateral margins, rounded tip, palate occupying about half of the rostrum length, oblong foramen and crossbar complete; (2) large lanceolate avicularia with lobate edge at half distal part (Fig 12B and 12D), placed distolaterally to the orifice and oriented proximally, palate broad, occupying about three-quarters of the rostrum length, foramen oval, crossbar complete; 1–2 pseudopores adjacent to the avicularia, rostrum spatulate, with crenulated margins (Fig 12A) in younger and strongly serrated margins (Fig 12B) in older zooids. Ovicells globose (Fig 12D), surrounded by an adjacent frontal wall; ectooecium with 12–27 medium-sized pseudopores (0.005–0.007–0.0013 mm wide; n = 30; SD = 0.001 mm) distributed through the entire ovicell surface. Secondary calcification around the primary orifice in ovicelled zooids forms a hood in the distal margin of the ovicell (Fig 12D).

#### Remarks

As like *P*. *ligulata* comb. nov., *P*. *serrula* belongs to the *P*. *spathulata–P*. *areolata* complex, thus having avicularia with two morphologies: narrow–elongated and large–spatulated. Species of this complex, however, can be distinguished by features of the primary orifice, avicularia ornamentation, and ovicells. *Parasmittina serrula* differs from *P*. *ligulata* comb. nov., *P*. *spathulata*, and *P*. *areolata* by having up to 6 oral spines (up to 4 in all other species), and large spatulate avicularium with strongly serrated margins (with smoother margins in all other species).

First described based on specimens from Hawaii, in the Central Pacific [2], *P*. *serrula* was already recorded from coral reefs of Belize, and experimental panels from Jamaica [3, 37] in the Caribbean and several localities from the Western and Central Pacific [2]. Although most reports of *P*. *serrula* from the Western and Central Pacific differ from the type specimens in characters of the primary orifice (Table 7), specimens from SW Atlantic studied here showed all morphological and morphometric characters provided by Soule & Soule [2] (Tables 6 and 7) to *P*. *serrula*. Its unusual geographical distribution was already related to hull shipping through the Panama Canal [16] and to an opportunistic behavior of the species [37].

Among congeners, *P*. *serrula* is very similar to *Parasmittina luteoserrula*, recently described by Winston & Jackson [13] based on specimens from the Caribbean. Winston & Jackson [17] compared *P*. *luteoserrula* with *P*. *serrula* and indicated differences in the zooid sizes (indicated incorrectly as smaller in *P*. *luteoserrula* than in *P*. *serrula*, but zooids in the former species are actually larger, as shown in Table 6), large avicularia with a more fluted rostrum, and pores occupying more than half of the frontal surface of the ovicell. However, as previously discussed (see remarks of *P*. *alba*, for example), much of these variations may be related to different colonial and zooidal stages of development rather than different species. Thus, we consider a species complex involving *P*. *serrula* and *P*. *luteoserrula*, but additional studies including specimens from the current distributional range of *P*. *serrula* are needed.

#### Distribution

Central Pacific: Hawaii [3]; SW Atlantic: Brazil (present study).

### *Parasmittina simpulata* Winston, Vieira & Woollacott, 2014

**Fig 13:**
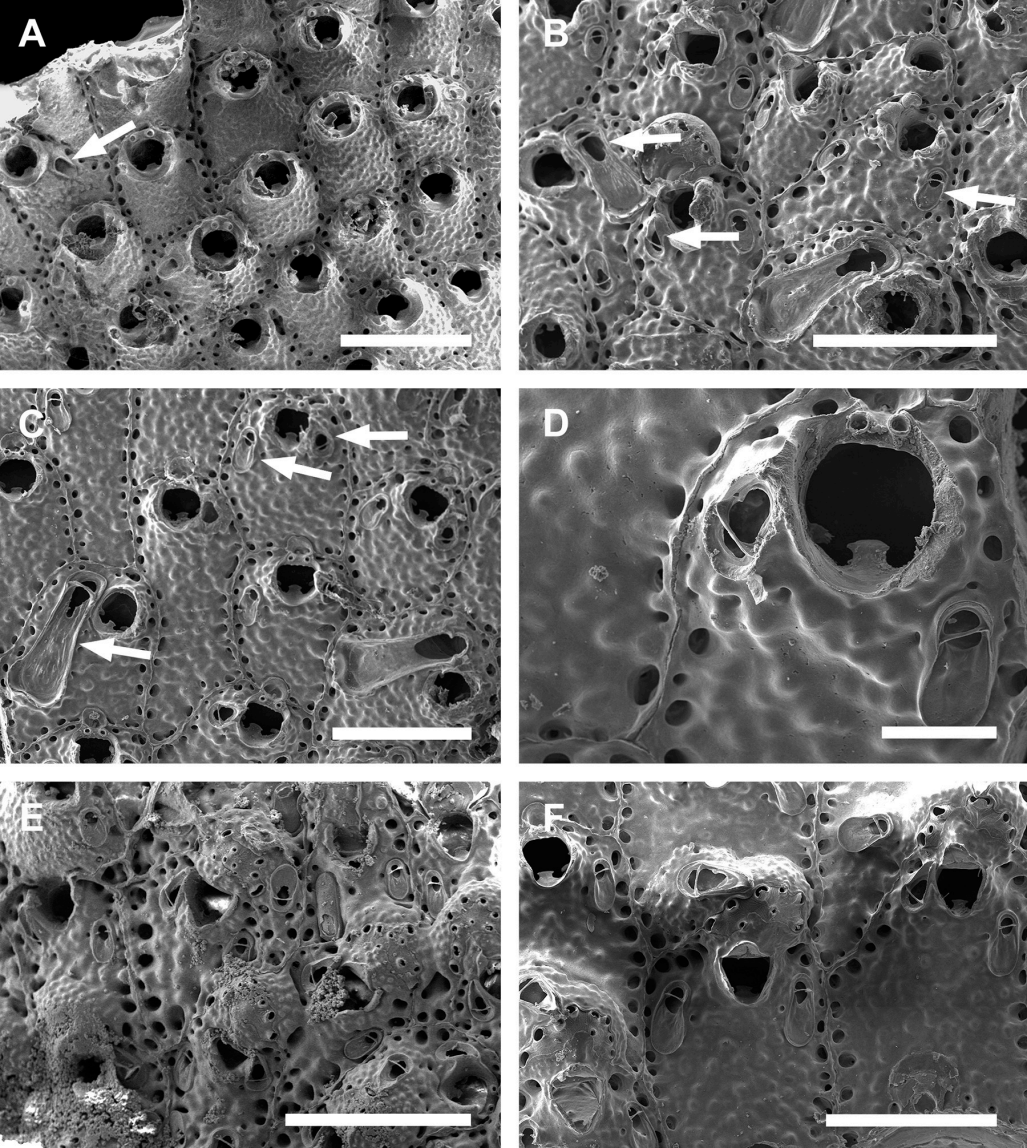
*Parasmittina simpulata* Winston, Vieira & Woollacott, 2014. (A) UFBA 365, (B–F) UFBA 5324, Bahia, Brazil. Arrows indicating avicularia. (A) autozooids at the growing edge of the colony showing the origin of the small avicularia, (B) group of autozooids with small and large avicularia, (C) detail of autozooid showing small and large avicularia, (D) detail of primary orifice showing small avicularia. (E) group of ovicelled zooids. (F) detail of ovicelled zooids showing secondary calcification around the ovicells. Scale bars: A, E = 500 μm; D = 100 μm; B = 200 μm; C, F = 400 μm.

(Fig 13; Tables 8, 10)

**Table 8:** Morphometric data of *P*. *simpulata*, *P*. *parsevaliformi*s and *P*. *barbadensis*. Values given in millimeters and represented as minimum–median–maximum (number of zooidal measures). Avicularia abbreviations: Av1 = small subtriangular; Av2 = small oblong; Av3 = large spatulate.

Character	Taxa / Reference / Specimen			
	*P*. *simpulata /* Winston *et al*. (2014)	*P*. *simpulata /* Present study / UFBA 5324	*P*. *parsevaliformi*s / Soule & Soule (1973)	*P*. *barbadensis /* Winston & Woollacatt (2009)
**ZL**	0.450–0.585–0.684 (6)	0.332–0.544 0.818 (15)	0.500–0.600–0.700 (10)	0.655–0.804–0.892 (6)
**ZW**	0.324–0.360–0.396 (6)	0.255–0.396–0.634 (15)	0.320–0.385–0.480 (10)	0.400–0.510–0.637 (6)
**OL**	0.090–0.111–0.126 (6)	0.081–0.0.097–0.127 (15)	0.110–0.127–0.160 (10)	0.127–0.141–0.155 (6)
**OW**	0.090–0.099–0.108 (6)	0.084–0.100–0.110 (15)	0.100–0.106–0.110 (10)	0.127–0.133–0.146 (6)
**Av1L**	0.162–0.447–0.612 (6)	0.086–0.131–0.188 (15)	0.140–0.168–0.200 (10)	0.127–0.133–0.146 (6)
**Av1W**	0.108–0.129–0.144 (6)	0.033–0.057–0.069 (15)	0.080–0.092–0.100 (10)	0.055–0.074–0.091 (6)
**Av2L**	0.126–0.180–0.270 (6)	0.097–0.124–0.160 (15	0.080–0.111–0.140 (10)	0.127–0.176–328 (6)
**Av2W**	0.054–0.081–0.144 (6)	0.039–0.046–0.056 (15)	0.040–0.053–0.060 (10)	0.055–0.086–0.200 (6)
**Av3L**	–	0.302–0.451–0.553 (8)	0.310–0.360–0.390 (10)	–
**Av3W**	–	0.100–0.126–0.156 (8)	0.120–0.174–0.200 (10)	–
**OvL**	–	0.199–0.226–0.254 (14)	0.200–0.215–0.240 (4)	0.237–0.267–0.291 (6)
**OvW**	–	0.225–0.270–0.346 (14)	0.240–0.245–0.260 (4)	0.291–0.340–0.382 (6)

*Parasmittina simpulata* Winston *et al*. 2014: 200, fig 39A–F [12].

*Parasmittina spathulata*: Almeida *et al*. 2015: 4 (in part) [26]. Non *Escharella jacotini* var. *spathulata* Smitt, 1873: 60 [42].

? *Parasmittina parsevaliformis* Soule & Soule 1973: 412 [2].

? *Parasmittina barbadensis* Winston & Woollacott 2009: 262 [38].

#### Material examined

UFBA 209, 716, 5304, 5313–5315, 5324, 5445, UFPE 831–832, Costa dos Coqueiros, Camaçari, Bahia, Brazil, 25–30 m, coll. 1993–2004; UFBA 365, Salvador, Bahia, Brazil, 0–25 m, coll. 05/04/2012; UFBA 952, 959, 2268, Baia de Todos os Santos, Bahia, Brazil, 0–48 m, coll. 1997–2006; UFBA 3110, 3112, 3116, Costa do Dendê, Bahia, Brazil, coll. 2012; UFBA 5325, Banco Charlotte, Bahia, Brazil, 35 m, coll. 03/1996.

#### Description

Colony encrusting, primarily unilaminar (Fig 13A). Autozooids (Fig 13A) initially subrectangular to subquadrangular, limited by slightly raised lateral walls, with a single row of 20–31 marginal pores (0.007–0.019–0.0040 mm in diameter; n = 30; SD = 0.007 mm). Frontal wall rugose and nodular (Fig 13B, 13C). Primary orifice (Fig 13D) elliptical, slightly longer than wide, distal with 4–6 well-spaced minute denticles, up to 3 oral spines, lyrula narrow (0.017–0.031–0.038 mm wide; n = 15; SD = 0.005 mm), a pair of hooked condyles with coarsely serrated margins. Secondary orifice initially developed as two short lateral projections (Fig 13C), sometimes forming a broad U-shaped pseudosinus (Fig 13F). Adventitious avicularia variable in shape and size (Fig 12B–12F), with three morphologies: (1) small, subtriangular, avicularia (Fig 13D), placed laterally at one side of the orifice, distally oriented, rostrum subtriangular, smooth at its lateral margins, pointed tip, palate narrow, subtriangular foramen, and crossbar complete; (2) small, oblong to spatulate avicularia (Fig 13D) single or paired, placed below the secondary orifice, proximally oriented, smooth at its lateral margins, rounded tip, palate occupying about three-quarters of the rostrum length, oval foramen and crossbar complete; (3) large, spatulate avicularia (Fig 13B and 13C), placed laterally at one side of the orifice, oriented proximally, with rostrum oblong to spoon-shaped arched upwards, palate broad occupying three-quarters of the rostrum length, foramen subtriangular to elliptical, with smooth margins and crossbar complete, with 2–3 pseudopores adjacent to the avicularia. Secondary calcification of the frontal wall can place avicularia randomly scattered on the frontal wall (i.e., around the orifice, in a suboral position, and on the ovicell, Fig 13E–13F). Ovicells globose, densely surrounded by adjacent frontal wall (Fig 13E); ectooecium with 8–10 large pseudopores (0.007–0.013–0.026 in diameter; n = 30; SD = 0.004 mm). Secondary calcification around the primary orifice in ovicelled zooids forms a hood in the distal margin of the ovicell (Fig 13F).

#### Remarks

*Parasmittina simpulata* was originally described based on a single infertile colony from southeastern Brazil, being diagnosed by having a primary orifice with distal denticles, serrated condyles, proximolateral small avicularia oval and subtriangular and large avicularia spatulate [12]. All these characters are observed in specimens from coral reefs and experimental plates of tiles from northeastern Brazil analyzed in this study, adding that our colonies have ovicell with large pseudopores (Fig 13E and 13F). Also, we observed that avicularia development and ovicell calcification vary between younger (Fig 13A and 13B) and older (Fig 13C and 13E) colonies.

*Parasmittina simpulata* is considered very similar to the Caribbean *P*. *barbadensis* Winston & Woollacott, 2009 [38], differing only in the large avicularia (absent in *P*. *barbadensis*) and larger zooids of *P*. *barbadensis* [12]. Unfortunately, *P*. *barbadensis* was also described based on a single colony fragment, preventing more comparisons. *Parasmittina simpulata* and *P*. *barbadensis* also share similarities in the frontal calcification, primary and secondary orifices, and avicularia with *P*. *parsevaliformis* Soule & Soule, 1973 [2] described based on specimens from Hawaii, except for the large avicularium (absent in *P*. *barbadensis*) (Table 8). Soule & Soule [2] described differences in avicularia morphology and secondary calcification in the orifice and ovicell pending the colonial development of *P*. *parsevaliformis*. We also found morphological variations in the avicularia development and ovicell calcification when compared younger (Fig 13A and 13B) and older (Fig 13E and 13F) colonies. Also, our values comprise morphometric variations seen in the three taxa–*P*. *simpulata*, *P*. *barbadensis* and *P*. *parsevaliformis* (Table 8). Thus, we have some evidence that the absence of avicularia in specimens originally attributed to *P*. *simpulata* and *P*. *barbadensis*, respectively, as well as the morphometric variations, can be related to the ontogenetic stage of the studied specimens.

Biogeographically, it is unlikely that *P*. *simpulata* (SW Atlantic), *P*. *barbadensis* (Caribbean), and *P*. *parsevaliformis* (Central Pacific) may represent a single species. On the other hand, *P*. *parsevaliformis* was described based on specimens that were encrusting a variety of substrata including rocks, corals, shells and cement [2] and material studied here include colonies from coral reefs and artificial substrata (experimental plates of tiles). Thus, the association with artificial substrata could suggest that a single species of *Parasmittina* may be dispersing worldwide, resulting in isolated geographical records. However, until more data on these taxa are known, we choose to keep them as distinct species.

#### Distribution

SW Atlantic: Brazil [12]; present study).

### *Parasmittina winstonae* Liu in Liu, Yin & Ma, 2001

**Fig 14:**
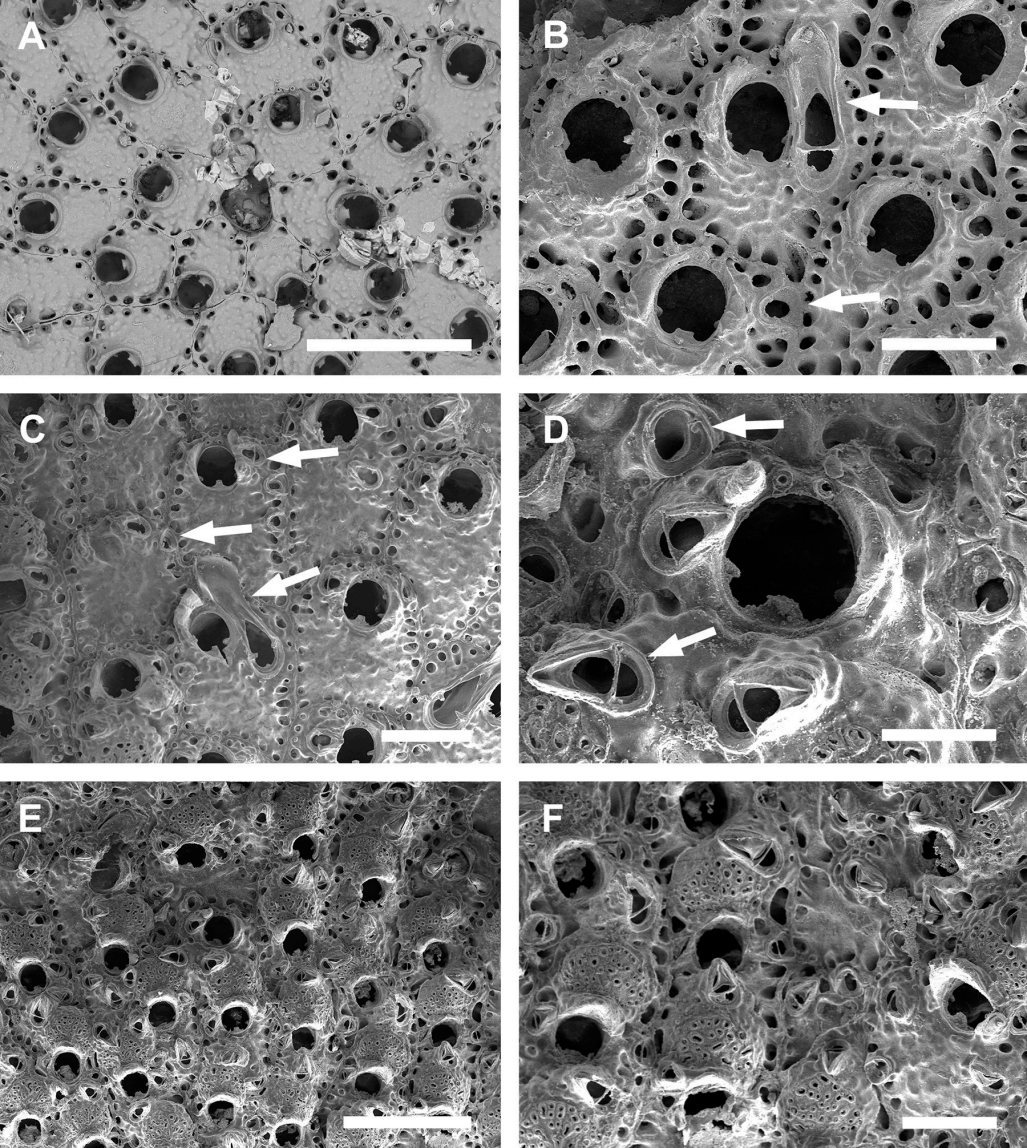
*Parasmittina winstonae* Liu in Liu, Yin & Ma, 2001. (A) UFBA 5321, (B) UFBA 5320, (C–F) UFBA 5316, Bahia, Brazil. Arrows indicating avicularia. (A) group of young autozooids, (B) detail of autozooid showing small and large avicularia, (C) group of autozooids with small and large avicularia, (D) detail of orifice, (E) group of ovicelled zooids, (F) detail of ovicelled zooids with showing secondary calcification. Scale bars: A, E = 500 μm; B, F = 200 μm; C = 250 μm; D = 100 μm.

(Fig 14; Tables 9, 10)

**Table 9:** Morphometric data of specimens here assigned to *P*. *winstonae*. Values given in millimeters and represented as minimum–median–maximum (number of zooidal measures). Avicularia abbreviations: Av1 = small subtriangular; Av2 = small spatulate; Av3 = large spatulate.

Character	Reference					
	Liu *et al*. (2001)	Present study / UFBA 5316	Tilbrook (2006)	Taylor & Tan (2015)	Winston *et al*. (2014)	Almeida *et al*. (2018)
**ZL**	0.440–0.574–0.660 (10)	0.337–0.412–0.514 (10)	0.450 (?)	0.320–0.450 (?)	0.306–0.360–0.396 (6)	0.344–0.396–0.561 (15)
**ZW**	0.280–0.327–0.420 (10)	0.196–0.307–0.367 (10)	0.300 (?)	0.220–0.330 (?)	0.216–0.240–0.270 (6)	0.160–0.278–0.358 (15)
**OL**	0.140–0.150–0.160 (10)	0.087–0.102–0.115 (8)	0.110 (?)	0.100–0.110 (?)	0.081–0.090–0.099 (6)	0.113–0.117–0.134 (15)
**OW**	0.100–0.104–0.110 (10)	0.085–0.100–0.119 (8)	0.110 (?)	0.100 (?)	0.090–0.096–0.108 (6)	0.084–0.115–0.126 (15)
**Av1L**	0.102–0.120–0.144 (10)	0.069–0.093–0.132 (15)	-	0.090–0.120 (?)	0.108–0.113–0.126 (4)	0.090–0.093–0.116 (15)
**Av1W**	0.058–0.100–0.124 (10)	0.032–0.042–0.058 (15)	-	0.050–0.060 (?)	0.054–0.054–0.054 (4)	0.051–0.058–0.061 (15)
**Av2L**	0.062–0.0.98–124 (10)	0.041–0.054–0.076 (15)	-	0.050–0.060 (?)	-	0.051–0.068–0.083 (15)
**Av2W**	0.042–0.064–0.082 (10)	0.032–0.043–0.058 (15)	-	0.050–0.080 (?)	-	0.035–0.045–0.052 (15)
**Av3L**	0.270–0.280–300 (10)	0.386–0.415–0.444 (2)	-	0.350–0.400 (?)	-	0.237–0.312–0.360 (15)
**Av3W**	0.130–0.140–0.150 (10)	0.055–0.064–0.074 (2)	-	0.110–0.130 (?)	-	0.069–0.084–0.097 (15)
**OvL**	0.205–0.214–0.225 (10)	0.172–0.190–0.208 (15)	-	0.130–0.160 (?)	0.162–0.180–0.198 (6)	0.131–0.164–0.200 (15)
**OvW**	0.212–0.250–0.292 (10)	0.205–0.227–0.273 (15)	-	0.180 (?)	0.234–0.246–0.270 (6)	0.154–0.205–0.237 (15)

*Parasmittina winstonae* Liu in Liu, Yin & Ma, 2001: 801, pl 55, fig 1–7 [45].

*Parasmittina winstonae*: Tilbrook 2006: 156, pl 29K, fig 35A–C [8]; Taylor & Tan 2015: 19, fig 11A–L [57].

*Parasmittina loxoides* Winston *et al*., 2014: 202, fig 40A–D [12]; Almeida *et al*. 2018: 409, fig 5A–F [58].

#### Material examined

UFPE 545–554, UFBA 1619, 1621, 1622, 1627, 1628, 1652, 1661, 5319–5322, 5467–5495, Baia de Todos os Santos, Ilha de Itaparica, Itaparica, Bahia, Brazil, intertidal, coll. 2012–2015; UFBA 2852, Baía Todos os Santos, Porto da Barra, Salvador, Bahia, Brazil, 24 m, coll. 07/2017, UFBA 575, 2847–2849, Costa dos Coqueiros, Camaçari, Bahia, Brazil, 22–27 m, coll. 1997–2002, UFBA 697, 5003, Costa do Dendê, Bahia, Brazil, 30 m, coll. 2002; UFPE 641–644, UFBA 5316, Praia do Francês, Marechal Deodoro, Alagoas, Brazil, coll. 03/02/2003; UFPE 651, Jatiuca, Maceio, Alagoas, Brazil, coll. 15/10/2005; MZUSP Uncatalogued PE26, UFPE 579, Porto de Suape, Cabo de Santo Agostinho, Pernambuco, Brazil, coll. 01/08/2020; UFPE 621, 649 Barra de Catuama, Goiana, Pernambuco, Brazil; UFBA 5323, Pontas de Pedra, Goiana, Pernambuco, Brazil, intertidal, coll. 20/02/2015, UFPE 343, 645–646, Porto do Cabedelo, Cabedelo, Paraíba, Brazil, intertidal, coll. 27/09/2009; UFPE 274, Saco da Pedra, coll. 16/09/2004; UFPE 340, Amores, 22/09/2006; UFBA 5017, 5160, 5167, 5212, 5262, 5276, 5293, 5405, 5421, 5459, 5496, 5499, 5511, 5513, Baia de Todos os Santos, Salvador, Bahia, Brazil, 3–5 m, coll. 2012, MZUSP 887, Santos, São Paulo, Brazil.

#### Description

Colony encrusting, unilaminar. Autozooids (Fig 14A) subrectangular to polygonal, limited by slightly raised lateral walls, with a single row of 25–30 large marginal pores (0.009–0.025–0.042 mm in diameter; n = 30; SD = 0.008 mm). Frontal wall slightly rugose and nodular. Primary orifice (Fig 14B) elliptical, slightly longer than wide, anter smooth, 1–2 oral spines, lyrula narrow (0.020–0.041 mm wide), and a pair of hook-shaped condyles with coarsely serrated tips. Secondary orifice low (Fig 14D), forming two lateral short projections. Adventitious avicularia variable in shapes and sizes (Fig 14B and 14F), with three morphologies: (1) small, subtriangular avicularia (Fig 14C and 14D), placed laterally to the orifice and distally oriented in autozooids, with a suboral or randomly arranged in ovicelled and adjacent zooids (Fig 14E–14F), rostrum subtriangular, smooth at its lateral margins, pointed tip, palate narrow, elliptical foramen and crossbar complete; (2) small, spatulate avicularia (Fig 14B and 14C), placed o zooidal margins, commonly replacing a marginal pore; (3) large, spatulate avicularia (Fig 14B and 14C), placed laterally at one side of the orifice, oriented distally, palate broad occupying half of the rostrum length, foramen subtriangular to elliptical, with smooth margins and crossbar complete, with 2–3 pseudopores adjacent to the avicularia. Ovicell (Fig 14E–14F) densely surrounded by adjacent frontal wall, soon becoming immersed in the frontal calcification; ectooecium with 20–32 medium-sized pseudopores (0.004–0.016 mm in diameter).

#### Remarks

Almeida *et al*. [58] redescribed *P*. *loxoides* based on specimens from Brazil. They characterized it by having subrectangular to polygonal autozooids with large marginal pores (Fig 14A), 1–2 oral spines (Fig 14B and 14D), narrow and short lyrula, condyles with serrated margins, and large avicularia distally directed. However, all these characteristics are also seen in *P*. *winstonae*, first described based on specimens from China by Liu *et al*. [45]. Moreover, no morphometric differences were seen among specimens from Brazil and China (Table 9) (see also Tilbrook [8, 57]. Thus, here we consider *P*. *winstonae* as the senior synonym of *P*. *loxoides*.

Among all congeners, *P*. *winstonae* most resembles *P*. *californica* (Robertson, 1908), *P*. *collifera* (Robertson, 1908) and *P*. *regularis* Soule & Soule, 2002 in having three types of avicularia–lateral, marginal, and large distally directed. However, whereas the large avicularium of *P*. *winstonae* is spatulate with a rounded tip, that of *P*. *californica*, *P*. *collifera* and *P*. *regularis* is subtriangular with an acute rostrum.

Like other species described here, *P*. *winstonae* haves an unusual geographic distribution in the Western and Central Pacific and SW Atlantic [8, 12, 45, 57, 58]. Interestingly, Chinese and Brazilian specimens of *P*. *winstonae* showed a common association with bivalve shells of the genus *Pinctada* Röding, 1798 [45, 58]. Colonies from Brazil studied here are also from artificial substrata, and specimens from Malaysia studied by Taylor & Tan [57] were encrusting shells on a rope hanging from a jetty. Therefore, passive dispersal may be related to the current distributional range of *P*. *winstonae*.

#### Distribution

Western and Central Pacific: China, Islands Solomon and Malaysia [8, 45, 57]; SW Atlantic: Brazil [12, 58] present study.

### *Parasmittina falciformis* sp. nov.

urn:lsid:zoobank.org:act:82A01897-244E-4B06-8A18-600FA9E6F9C9

(Fig 15, Table 10)

**Fig 15:**
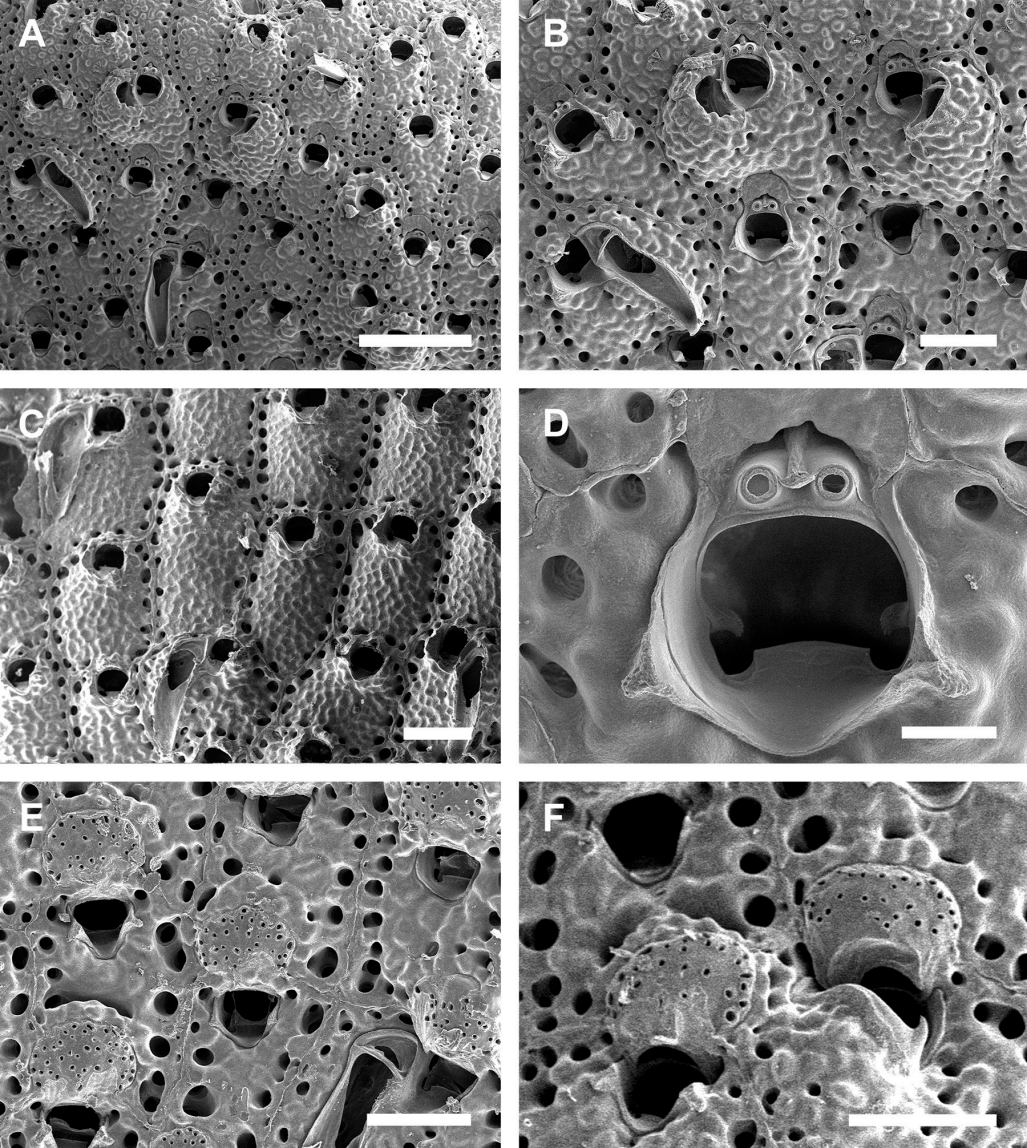
*Parasmittina falciformis* sp. nov. (A–B, D–F) UFBA 714, holotype, (C) UFBA 953, paratype, Bahia, Brazil. Arrows indicating avicularia. (A) group of young autozooids, (B) detail of autozooid showing small and large avicularia, (C) group of autozooids showing large avicularia, (D) detail of primary orifice, (E) group of ovicelled zooids, (F) detail of ovicells. Scale bars: A = 250 μm; B, E, F = 200 μm; C = 500 μm; D = 50 μm.

**Table 10:** Diagnostic characters of *Parasmittina* species, with respective type localities, and based on features of the primary orifice (distal margin ornamentation, number of spines, condyles profile/condyles margin ornamentation, and lyrula size), small avicularia morphologies, large avicularia morphologies, and number of ovicell pores. Symbols and abbreviations: (Isl.) = Island, (*) = type specimen lost, (**) = species only with vicarious avicularia, (?) = unknown states, (-) = absent, (S) = spines, (L) = lyrula and orifice width ratio.

Species	Type locality	Primary orifice				Small avicularia			Large avicularia	Ovicell pores
		Distal margin	S	Condyles	L	Distolateral	Proximolateral	Marginal		
*P*. *abrolhosensis* Ramalho *et al*., 2018	Bahia, Brazil	smooth	3–4	robust/serrated	0.25	-	elongate/ subtriangular	subtriangular/ elliptical	spatulate	?
*P*. *aculeata* Tilbrook, 2006	Solomon Isl., Oceania	smooth	1–2	thin/smooth	0.50	sublanceolate	-	-	-	?
*P*. *acuta* (Canu & Bassler, 1929)	Philippines	?	?	thin/?	?	-	elongate	?	?	?
*P*. *alanbanneri* Soule & Soule, 1973	Hawaii, Pacific USA	smooth	3–6	?	0.50	-	subtriangular	-	sublanceolate	20 or >
*P*. *alaskensis* Osburn, 1952	Alaska, USA	smooth	2	?	?	-	subtriangular/ elliptic	-	-	?
*P*. *alba* Ramalho, Muricy & Taylor, 2011	Rio de Janeiro, Brazil	smooth	1–2	thin/smooth	0.50	subtriangular	subtriangular/ spatulate	-	spatulate	> 10
*P*. *aleutensis* Soule & Soule, 2002	Alaska, USA	smooth	2–3	thin/smooth	0.33	-	-	subtriangular/ oblong	-	2–3
*P*. *alitis* Winston & Jackson, 2021	Jamaica, Caribbean	beaded	3–4	robust/serrated	0.33	-	elongate/ subtriangular	-	elongate/ sublanceolate	25–29
*P*. *amazonensis* Ramalho & Moraes in Ramalho *et al*. 2021	Maranhão, Brazil	smooth	5–6	?	0.75	elliptical	elliptic	elliptic	-	20 or >
*P*. *aotea* (Brown, 1952)	New Zealand	?	3	?	0.25	-	subtriangular	-	sublanceolate	?
*P*. *areolata* (Canu & Bassler, 1927)	Hawaii, Pacific USA	smooth	2	?	0.12	subtriangular	elongate	-	spatulate	32
*P*. *atypica* (Powell, 1967)	New Zealand	?	2–5	?	0.75	subtriangular	-	subtriangular	-	?
*P*. *aviculifera* Soule & Soule, 2002	California, Pacific USA	smooth	1–3	robust/serrated	0.50	-	oblong	oblong/ subtriangular	subtriangular	18 or >
*P*. *baccula* Hayward & Winston, 2011	Antarctic, USA	beaded	1–2	robust/serrated	0.50	-	oblong/ spatulate	-	sublanceolate	24–27
*P*. *barbadensis* Winston & Woollacott, 2009	Barbados, Caribbean	beaded	1–2	robust/serrated	0.25	subtriangular	oblong/ spatulate	oblong/ spatulate	spatulate	8–10
*P*.* betamorphaea *Winston, 2005* *	Florida, Atlantic USA	smooth	2	thin/smooth	0.50	-	elliptic/ subtriangular	-	spatulate	?
*P*. *bimucronata* (Hincks, 1884a)	Myanmar, Andaman Sea	smooth	1–2	thin/smooth	0.25	-	subtriangular/ oblong	oblong	sublanceolate	20–37
*P. *californica* (Robertson, 1908)	California, Pacific USA	?	?	?	?	-	subtriangular	subtriangular/ oblong	sublanceolate	20 >
*P*. *californiensis* (Robertson, 1908)	California, Pacific USA	?	2–4	?	?	-	subtriangular/ sublanceolate	subtriangular	?	?
*P*. *circinanata* Liu in Liu *et al*., 2001	China Sea	smooth	2	robust/serrated	0.25	subtriangular	-	spatulate	-	?
*P*. *circularis* Soule & Soule, 1973	Hawaii, Pacific USA	smooth	2	robust/?	0.25	-	subtriangular/ elliptic	subtriangular/ elliptic	spatulate	?
*P. *collifera* (Robertson, 1908)	California, Pacific USA	smooth	1–2	?	0.33	-	-	subtriangular	-	8–14
*P*. *crosslandi* (Hastings, 1930)	Taboga Isl., Pacific Panama	?	2–3	?	0.75	-	elongate	-	sublanceolate	20 or >
*P*. *cyclops* Winston & Jackson, 2021	Jamaica, Caribbean	smooth	2–3	thin/smooth	0.50	subtriangular	-	-	-	10 or >
*P*. *delicatula* (Busk, 1884)	Hawaii, Pacific USA	?	?	thin/smooth	?	-	subtriangular/ oblong	subtriangular/ oblong	spatulate	-
*P*. *dentigera* (Harmer, 1957)	Indonesia	beaded	2–3	?	?	-	subtriangular	subtriangular	?	?
*P*. *dependeo* Tilbrook, 2006	Solomon Isl., Oceania	smooth	2	robust/serrated	0.25	-	-	oblong/ spatulate	-	?
*P*. *distincta* Ramalho *et al*., 2018	Bahia, Brazil	beaded	2–4	robust/?	0.50	-	oblong/ subtriangular	-	sublanceolate	?
*P*. *dolabrata* Soule & Soule, 1973	Galápagos Isl.	smooth	1–3	robust/serrated	0.33	subtriangular	oblong/ spatulate	oblong/ spatulate	hatchet-shaped	?
*P*. *dubitata* Hayward, 1980	Argentina	?	1–3	robust/?	0.50	-	oblong	oblong/ spatulate	subtriangular	12
*P*. *eccentrica* Winston & Jackson, 2021	Jamaica, Caribbean	smooth	2–3	robust/serrated	0.33	-	elongate	subtriangular/ elliptic	hatchet-shaped/ elongate	20 or >
*P*. *echinata* (Canu & Bassler, 1928b)	Gulf of Mexico	?	?	?	0.25	-	subtriangular	-	-	?
*P*. *egyptiaca* (Waters, 1909)	Egypt, Red Sea	smooth	1–2	robust/serrated	0.33	-	oblong/ spatulate	-	sublanceolate	20 or >
*P*. *emersoni* Soule & Soule, 1973	Hawaii, Pacific USA	smooth	2	robust/?	0.33	-	subtriangular	subtriangular	-	?
*P*. *erecta* Gordon & d’Hondt, 1997	New Caledonia	smooth	6	?	?	-	subtriangular/ sublanceolate	subtriangular	sublanceolate	44
*P*. *exasperatrix* d’Hondt, 1986	New Caledonia	smooth	3	?	?	subtriangular	elongate/ sublanceolate	subtriangular	-	?
*P*. *exiguiuncinata* Tilbrook, 2006	Solomon Isl., Oceania	smooth	1–3	robust/serrated	0.33	-	elongate/ subtriangular	subtriangular	-	?
*P*. *fistulata* (Harmer, 1957)* *	Makassar Strait, Indonesia	smooth	1	thin/smooth	0.33	-	subtriangular/ oblong	oblong	spatulate	?
*P*. *floridana* Winston, 2005	Florida, Atlantic USA	beaded	2–3	thin/serrated	0.50	-	subtriangular/ oblong	subtriangular/ oblong	-	16
*P*. *fraseri* Osburn, 1952	Clarion Isl., Mexican Pacific	smooth	3–5	?	0.75	oblong/ elliptic	-	oblong/ elliptic	-	30
*P*. *galerita* Ryland & Hayward, 1992	Heron Isl., Australia	smooth	?	robust/serrated	0.33	subtriangular	oblong/ spatulate	oblong/ spatulate	spatulate	20
*P*. *glabra* Gordon & d’Hondt, 1997	New Caledonia	smooth	7–8	?	0.33	-	subtriangular	-	-	40
*P*. *glomerata* (Thornely, 1912)	Mascarene Isl., Mauritius	beaded	1–2	?	?	-	oblong	oblong	spatulate	?
*P*. *hanzawae* Kataoka, 1960	Japan Sea	?	2	?	?	-	subtriangular	subtriangular/ elliptic	-	?
*P*. *hastingsae* Soule & Soule, 1973	Hawaii, Pacific USA	smooth	2–3	?	?	-	oblong	-	subtriangular/ sublanceolate	?
*P*. *ilioensis* Soule & Soule, 1973	Hawaii, Pacific USA	smooth	2–3	thin/smooth	0.33	subtriangular	subtriangular/ oblong	oblong/ subtriangular	sublanceolate	?
*P*. *inalienata* Tilbrook, 2006	Solomon Isl., Oceania	smooth	1–2	thin/smooth	0.25	-	oblong	oblong	-	15
*P*. *indiginella* Winston, 2016	Florida, Atlantic USA	smooth	5–6	?	0.75	-	elliptical	elliptical	-	22–25
*P*. *kauaiensis* Soule & Soule, 1973	Hawaii, Pacific USA	smooth	2	thin	0.25	-	oblong/ subtriangular	oblong/ subtriangular	sublanceolate	?
*P*. *labellum* (Canu & Bassler, 1928b)	Gulf of Mexico	?	2	?	0.25	subtriangular	-	subtriangular/ oblong	spatulate	?
*P*. *latiavicularia* (Kirkpatrick, 1888)	Mauritius, Indian Ocean	smooth	1–2	?	?	subtriangular	-	oblong/spatulate	spatulate	10 or >
*P*. *lavela* Soule & Soule, 2002	Peninsula de La Guajira, Colombia	smooth	2	thin/smooth	0.50	subtriangular	subtriangular	-	-	12 >
*P*. *leviavicularia* Soule & Soule, 1973	Hawaii, Pacific USA	smooth	2	?	0.50	subtriangular/ sublanceolate	oblong	oblong	-	14–24
*P*. *livingstonei* (Powell, 1967)	New Zealand	?	5–7	?	0.75	-	-	subtriangular	-	?
*P*. *ligulata* comb. nov. (Ridley, 1881)	Espírito Santo, Brazil	smooth	2–4	robust/serrated	0.25	-	elongate	elliptic	elongate/ sublanceolate	20–26
*P*. *longirostrata* Liu in Liu *et al*., 2001	China Sea	smooth	2–4	robust/serrated	0.50	-	oblong/elongate	-	sublanceolate	20–26
*P*. *loxoides* Winston *et al*., 2014	Rio de Janeiro, Brazil	smooth	1–2	robust/serrated	0.25	subtriangular	-	spatulate	spatulate	25 or >
*P*. *luteoserrula* Winston & Jackson, 2021	Jamaica, Caribbean	beaded	3–4	robust/serrate	0.25	-	elongate	-	noodle tong/ elongate	19–28
*P*. *macginitiei* Soule & Soule, 2002	Alaska, USA	smooth	2	thin/smooth	0.33	-	subtriangular	oblong/ spatulate	-	3
*P*. *margaritata* Hayward, 1988	Mauritius, Indian Ocean	smooth	3–4	thin/smooth	0.50	-	oblong	oblong/ subtriangular	sublanceolate	-
*P*. *marsupialis* (Busk, 1884)	Hawaii, Pacific USA	?	?	?	0.25	subtriangular	oblong	oblong	-	?
*P*. *mauritiana* Hayward, 1988	Mauritius, Indian Ocean	smooth	1–4	thin/smooth	0.33	-	oblong	oblong	spatulate	?
*P*. *mexicana* Pouyet & Herrera-Anduaga, 1986	Gulf of Mexico	?	2	?	?	subtriangular	subtriangular	subtriangular	spatulate	15 or >
*P*. *multiaviculata* Souto *et al*., 2016	Madeira Isl.	smooth	1–2	thin/smooth	0.50	-	-	oblong/ spatulate	-	34
*P*. *munita* (Hincks, 1884b)	Australia	?	3	?	?	-	oblong	-	sublanceolate**	?
*P*. *murarmata* (Kirkpatrick, 1888)	Mauritius, Indian Ocean	?	?	?	0.50	sublanceolate	-	-	-	?
*P*. *nasuta* (Harmer, 1957)	Indonesia	?	3–6	?	?	-	elongate/ oblong	oblong/ subtriangular	spatulate	?
*P*. *natalensis* O’Donoghue, 1957	Umhlanga Rocks, Southern Africa-Indian	smooth	1–2	smooth	0.50	-	elliptic	-	-	?
*P*. *nitida* (Verrill, 1875)	Vineyard Sound, Atlantic USA	smooth	2–3	thin/?	0.25	-	subtriangular	oblong/spatulate/subtriangular	-	10 or >
*P*. *novella* Hayward & Cook, 1983	South Africa, Southern Africa-Indian	?	2–4	?	0.33	-	elliptic/ sublanceolate	-	sublanceolate/ noodle tong	?
*P*. *obstructa* (Waters, 1889)	Australia	?	2–3	?	0.33	sublanceolate	subtriangular	-	spatulate**	?
*P*. *oculinae* Winston, 2016	Florida, Atlantic USA	smooth	2	robust/smooth	0.33	-	elliptic/ subtriangular	-	-	34–44
*P*. *onychorrhyncha* Ryland & Hayward, 1992	Heron Isl., Australia	smooth	1	thin/smooth	0.50	-	-	oblong/ spatulate	spatulate	34
*P*. *ornata* (Thornely, 1912)	Mesh Skirt Bank, Indian Ocean	smooth	2	?	0.50	subtriangular	-	oblong	spatulate	?
*P*. *ovilirata* Tilbrook, 2006	Solomon Isl., Oceania	smooth	2	thin/smooth	0.33	subtriangular	subtriangular	subtriangular	spatulate	30 or >
*P*. *papulata* (Harmer, 1957)	Indonesia	smooth	?	thin/smooth	0.50	?	oblong/ subtriangular	oblong/ subtriangular	-	?
*P*. *paradicei* (Livingstone, 1926)	Great Barrier Reef, Australia	?	?	?	?	subtriangular	-	-	spatulate	10 or >
*P*. *parsevaliformis* Soule & Soule, 1973	Hawaii, Pacific USA	beaded	2–3	robust/serrated	0.33	subtriangular	oblong	oblong/ subtriangular	spatulate	10 or >
*P*. *parsevalii* (Audouin, 1826)	Gulf of Suez, Red Sea	beaded	1–2	robust/serrated	0.33	subtriangular	oblong	oblong/ subtriangular	subtriangular**	?
*P*. *parsevalioidea* Liu in Liu *et al*., 2001	China Sea	smooth	1–2	robust/serrated	0.50	subtriangular	-	oblong/ spatulate	spatulate	15 or >
*P*. *parsloeparsloei* Hayward & Parker, 1994	Australia	beaded	1	?	0.50	-	oblong	oblong	sublanceolate	20
*P*. *parvitatis* Tilbrook, 2006	Solomon Isl., Oceania	smooth	1	robust/serrated	0.25	-	oblong	oblong	spatulate	?
*P*. *parviuncinata* Soule & Soule, 1973	Hawaii, Pacific USA	smooth	2	?	?	-	elongate/ sublanceolate	subtriangular	-	25 or >
*P*. *pectinata* Hayward & Parker, 1994	Victoria, Australia	beaded	2–3	robust/serrated	0.50	-	oblong	-	sublanceolate	?
*P*. *pinctatae* Liu in Liu *et al*., 2001	China Sea	smooth	1–2	?	0.33	subtriangular	-	sublanceolate/oblong	spatulate	20 or >
*P*. *proximoproducta* Moyano, 1983	Chile	?	4	?	0.50	-	-	subtriangular	sublanceolate	?
*P*. *pugetensis* Soule & Soule, 2002	Puget Sound, Pacific USA	smooth	1–2	robust/serrated	0.25	-	-	spatulate/ subtriangular	sublanceolate	8 or >
*P*. *pyriformis* Seo, 2002	Ulsan, South Korea	smooth	1–3	?	?	-	subtriangular	-	subtriangular	?
*P*. *raigiformis* Soule & Soule, 1973	Hawaii, Pacific USA	smooth	2	robust/?	0.33	-	-	oblong/ subtriangular	spatulate/ sublanceolate	?
*P*. *raigii* (Audouin, 1826)	Gulf of Suez, Red Sea	beaded	1–2	thin/smooth	0.33	-	oblong	-	sublanceolate**	-
*P*. *raigioidea* Liu in Liu *et al*., 2001	China Sea	smooth	2	?	0.33	-	oblong	subtriangular	spatulate	18 or >
*P*. *recidiva* Hayward, 1988	Mauritius, Indian Ocean	smooth	3	?	0.50	-	sublanceolate	-	sublanceolate	?
*P*. *regularis* Soule & Soule, 2002	California, Pacific USA	beaded	1–2	robust/serrated	0.50	subtriangular	oblong	-	-	?
*P*. *rimula* Tilbrook, 2006	Solomon Isl., Oceania	smooth	1–2	thin/smooth	0.50	-	subtriangular	subtriangular	spatulate	?
*P*. *rostriformis* (Kirkpatrick, 1888)	Mauritius, Indian Ocean	beaded	4	?	?	-	elongate/ subtriangular	subtriangular	elongate/ sublanceolate	20 or >
*P*. *santacruzana* Soule & Soule, 2002	California, Pacific USA	smooth	?	robust/serrated	0.33	subtriangular	-	subtriangular/ oblong	sublanceolate**	7–9
*P*. *serrula* Soule & Soule, 1973	Hawaii, Pacific USA	beaded	3–6	robust/?	0.25	-	elongate	subtriangular	noodle tongs	12 or >
*P*. *serruloides* Harmelin *et al*., 2009	Lebanon, Mediterranean Sea	smooth	2–4	robust/serrated	0.33	-	oblong/ elongate	-	sublanceolate	20
*P*. *simpulata* Winston *et al*., 2014	Rio de Janeiro, Brazil	beaded	1–2	robust/serrated	0.25	subtriangular	oblong	oblong/ spatulate	spatulate	-
*P*. *solenosmilioides* Hayward & Parker, 1994	Investigator Strait, Australia	beaded	1–2	robust/?	0.33	-	subtriangular/ oblong	oblong	spatulate	15 or >
*P*. *spathulata* (Smitt, 1873)	Florida, Atlantic USA	?	2–4	?	0.50	-	elongate	-	spatulate	?
*P*. *spiculata* Gluhak *et al*., 2007	Taiwan, China Sea	?	?	?	?	-	elongate	-	elongate	24
*P*. *spondylicola* Harmelin *et al*., 2009	Lebanon, Mediterranean Sea	smooth	1	robust/smooth	0.75	subtriangular	oblong/ spatulate	-	spatulate	15–20
*P*. *subtubulata* (Harmer, 1957)	Indonesia	smooth	1–2	?	?	subtriangular	oblong	-	spatulate	?
*P*. *talismani* (Calvet, 1907)	Cape Verde, Africa	smooth	4–5	thin/smooth	0.75	oblong/elliptic	oblong/elliptic	oblong/elliptic	-	20–35
*P*. *tatianae* Denisenko, 2015	Chukchi Sea, Arctic	smooth	3–4	robust/serrated	0.33	-	subtriangular	-	-	?
*P*. *triangularis* (Mawatari, 1952)	Kii Peninsula, Japan Sea	?	?	?	?	subtriangular	subtriangular/ elongate	subtriangular	sublanceolate	?
*P*. *trianguliforma* Soule & Soule, 2002	Gulf of California, Pacific USA	?	2	?	0.75	-	subtriangular	-	-	20 or >
*P*. *trispinosa* (Johnston, 1838)	Berwick Bay, Gulf of Mexico	smoot	2–3	thin/smooth	0.25	-	-	oblong/ spatulate	subtriangular/ sublanceolate	2–4
*P*. *tropica* (Waters, 1909)	Egypt, Red Sea	?	2	?	?	-	elongate	-	sublanceolate	20
*P*. *trunculata* Tilbrook, 2006	Solomon Isl., Oceania	smooth	1–3	robust/serrated	0.12	-	elongate	-	spatulate	20 or >
*P*. *tubula* (Kirkpatrick, 1888)	Mauritius, Indian Ocean	?	6	?	?	-	oblong	-	-	?
*P*. *tubulata* Osburn, 1952	California, Pacific USA	?	?	robust/serrated	0.33	-	oblong	oblong/ subtriangular	spatulate	25–30
*P*. *turbula* Ryland & Hayward, 1992	Great Barrier Reef, Australia	?	1	robust/serrated	0.33	subtriangular	elongate	subtriangular/ elliptic	spatulate/ elongate	25 or >
*P*. *uncinata* Soule & Soule, 1973	Hawaii, Pacific USA	smooth	2–3	?	0.25	-	elongate	subtriangular	spatulate/ elongate	?
*P*. *variabilis* Liu in Liu *et al*., 2001	China Sea	smooth	1–2	robust/?	0.33	-	-	oblong	sublanceolate	20 or >
*P*. *veniliae* Winston & Jackson, 2021	Jamaica, Caribbean	beaded	1–2	robust/serrated	0.33	-	oblong	oblong/ subtriangular	sublanceolate	22–30
*P*. *winstonae* Liu in Liu *et al*., 2001	China Sea	smooth	1–2	robust/serrated	0.25	subtriangular	-	spatulate	spatulate	20 or >
*P*. *falciformis* sp. nov.	Bahia, Brazil	smooth	1–2	robust/serrated	0.50	subtriangular	-	-	sublanceolate	20–32

*Parasmittina munita*: Almeida *et al*. 2015: 4 [26].

Non *Smittia trispinosa* var. *munita* Hincks, 1884b: 284 [48]; Non *Smittina trispinosa* var. *munita*: Marcus, 1937: 108 [23]; Non *Smittina trispinosa* var. *munita*: Marcus, 1938: 44 [34]; Non *Parasmittina munita*: Vieira *et al*. 2008: 26 [25].

#### Material examined

*Holotype*: UFBA 714, Camaçari, Bahia, Brazil, 50 m, coll. 07/2004. *Paratypes*: UFBA 314, Costa do Descobrimento, Banco Charlotte, Bahia, Brazil, 35 m coll. 03/1996; UFBA 953, Baía de Todos os Santos, Bahia, Brazil, coll. 27/05/1997; UFBA 948, Baía de Todos os Santos, Bahia, Brazil, coll. 29/05/1997. *Additional specimens*: UFBA 5000, Banco Besnard, Espírito Santo, Brazil, coll. 04/1996; UFPE 3100–3101, Ilhas Rasas, Guarapari, Espírito Santo, Brazil, 11–15 m, coll. 27/03/2017; UFPE 3102–3105, Ilha Escalvada, Guarapari, Espírito Santo, Brazil, 15m, coll. 29/03/2017. UFPE 2599.1, Fortaleza, Ceara, Brazil, 24-27m. Coll. 1965–1966.

#### Etymology

Alluding to the profile of the large avicularium, subspatulate to sublanceolate, that leaves a falciform (hooked) outline.

#### Diagnosis

*Parasmittina* with elliptical orifice, smooth distal margin, lyrula occupying three quarters of the orifice width, and robust downward-facing condyles with serrated margin. Secondary orifice low, forming side flaps without hiding the lyrula. Adventitious avicularia subtriangular smaller and large subspatulate to sublanceolate, with hooked tip, placed distolaterally to the orifice and with rostrum oriented proximolaterally. Ovicell globose with 20–32 medium-sized pseudopores distributed through the entire ovicell surface.

#### Description

Colony encrusting, uni to multilaminar. Autozooids (0.322–0.432–0.584 mm length; n = 15; SD = 0.075 mm; 0.270–0.369–0.460 mm wide; n = 15; SD = 0.063 mm), (Fig 15A and 15B) subrectangular to subquadrangular, limited by slightly raised lateral walls, surrounded by a single row of 14–24 marginal pores (0.011–0.025–0.052 mm in diameter; n = 30; SD = 0.010 mm). Frontal wall with several rounded nodules (Fig 15A and 15B). Primary orifice (0.062–0.081–0.0.94 mm length; n = 15; SD = 0.009 mm; 0.087–0.098–0.105 mm wide; n = 15; SD = 0.004), (Fig 15D) elliptical, longer than wide, distal margin smooth, 1–2 oral spines, lyrula large (0.049–0.058–0.076 mm wide; n = 15; SD = 0.006 mm), comprising half of the orificial width, a pair of hooked condyles with serrated margins. Secondary orifice forming 2 proximolateral flaps resulting in a pseudosinus (Fig 15B and 15D), larger in ovicelled zooids (Fig 15E). Adventitious avicularia variable in shape and size (Fig 15B), with two morphologies: (1) small to moderate in size (0.154–0.169–0.184 mm in length; n = 3; SD = 0.014 mm; 0.052–0.064–0.078 mm in wide; n = 3; SD = 0.013 mm), subtriangular avicularia (Fig 15B), with a hooked tip and large foramen, placed distolaterally to the orifice, with rostrum oriented proximolaterally; (2) large avicularia (Fig 15B and 15C) (0.336–0.384–0.457 mm in length; n = 9; SD = 0.039mm; 0.077–0.083–0.088 mm in wide; n = 9; SD = 0.004 mm), reaching almost the entire zooidal length, profile subspatulate to sublanceolate, broad palate, occupying about half rostrum, foramen elliptical, rostrum subtriangular, with smooth margins, hooked tip and crossbar complete; 3–6 pseudopores adjacent to the avicularia, placed distolaterally to the orifice with rostrum oriented proximally. Ovicells (Fig 15E and 15F) (0.156–0.176–0.212 mm in length; n = 15; SD = 0.016mm; 0.153–0.198–0.240 mm in wide; n = 15; SD = 0.021 mm) globose, densely surrounded by adjacent frontal wall (Fig 15F); ectooecium with 25–30 medium-sized pseudopores (0.003–0.007–0.012 in diameter; n = 30; SD = 0.002 mm) distributed through the entire ovicell surface.

#### Remarks

Specimens of *P*. *falciformis* sp. nov. were previously attributed to *P*. *munita* by Almeida *et al*. [26] that is known from Australia [48]. However, it is distinguished from *P*. *falciformis* sp. nov. by the number of spines (3 in *P*. *munita* and up to 2 in *P*. *falciformis* sp. nov.) shape of pseudosinus (deep in *P*. *munita* and shallow in *P*. *falciformis* sp. nov.) and profile of the small adventitious avicularia (oblong in *P*. *munita* and subtriangular in *P*. *falciformis* sp. nov.).

Other specimens of *Parasmittina* from Brazil were also attributed to *Smittina trispinosa* var. *munita* [23, 33]. Although these specimens have avicularia similar to that of *P*. *falciformis* sp. nov., differences among these taxa include the number of oral spines (3 in specimens from Marcus and 2 in *P*. *falciformis* sp. nov.), secondary orifice (shorter in *P*. *falciformis* sp. nov.), pseudosinus (shallower in *P*. *falciformis*), and rostrum of the small adventitious avicularia (straight in specimens from Marcus and curved in *P*. *falciformis* sp. nov.). Moreover, specimens studied by Marcus [23, 34] have also a large interzooidal avicularia, not observed in *P*. *falciformis* sp. nov. Thus, here we consider that *Smittina trispinosa* var. *munita* described by Marcus [23, 34] represent a distinct taxon than *P*. *munita* and *P*. *facilformis* sp. nov.

The combination of primary orifice with large lyrula, smooth distally, 1–2 oral spines, serrated condyles with hooked tips, and two types of avicularia distinguishes *P*. *falciformis* sp. nov. from all congeners.

#### Distribution

SW Atlantic: Brazil (present study).

## Discussion

Although new species of *Parasmittina* from SW Atlantic have been described in the last years [10, 11, 12, 13, 59], we present the first review of historical records of the genus in the area. Additionally, to summarize the taxonomic knowledge regarding *Parasmittina* species worldwide, we compiled information on morphological features from the literature, mainly based on the original or subsequent descriptions of type specimens. This information is provided table with diagnostic characters of the *Parasmittina* species, including almost all living species. Thirteen taxa were not included as their original descriptions lack detailed morphological characterization and figures, type specimens were not found, and there is no characterization based on topotype specimens—*P*. *avicularissima* Gontar, 1982; *P*. *breli* d’Hondt & Mascarell, 2010; *P*. *cheilodon* (MacGillivray, 1869) *P*. *contraria* Seo, 1992; *P*. *jeffreysi* (Norman, 1876); *P*. *geometrica* (Kirkpatrick, 1890); *P*. *japonica* (Ortmann, 1890); *P*. *loxa* (Marcus, 1937); *P*. *projecta* (Okada & Mawatari, 1937); *P*. *protecta* (Thornely, 1905); *P*. *rouvillei* (Calvet, 1902); *P*. *soulesi* Scholz & Cusi, 1993 and *P*. *vacuramosa* Lu, Nie & Zhong in Lu, 1991. Thus, we present diagnostic features of the primary orifice (distal margin ornamentation, number of spines, condyles profile and ornamentation, and lyrula and orifice width ratio), avicularia morphologies, ovicell size, and number of ovicell pores of 120 *Parasmittina* species (Table 10).

**Fig 16:**
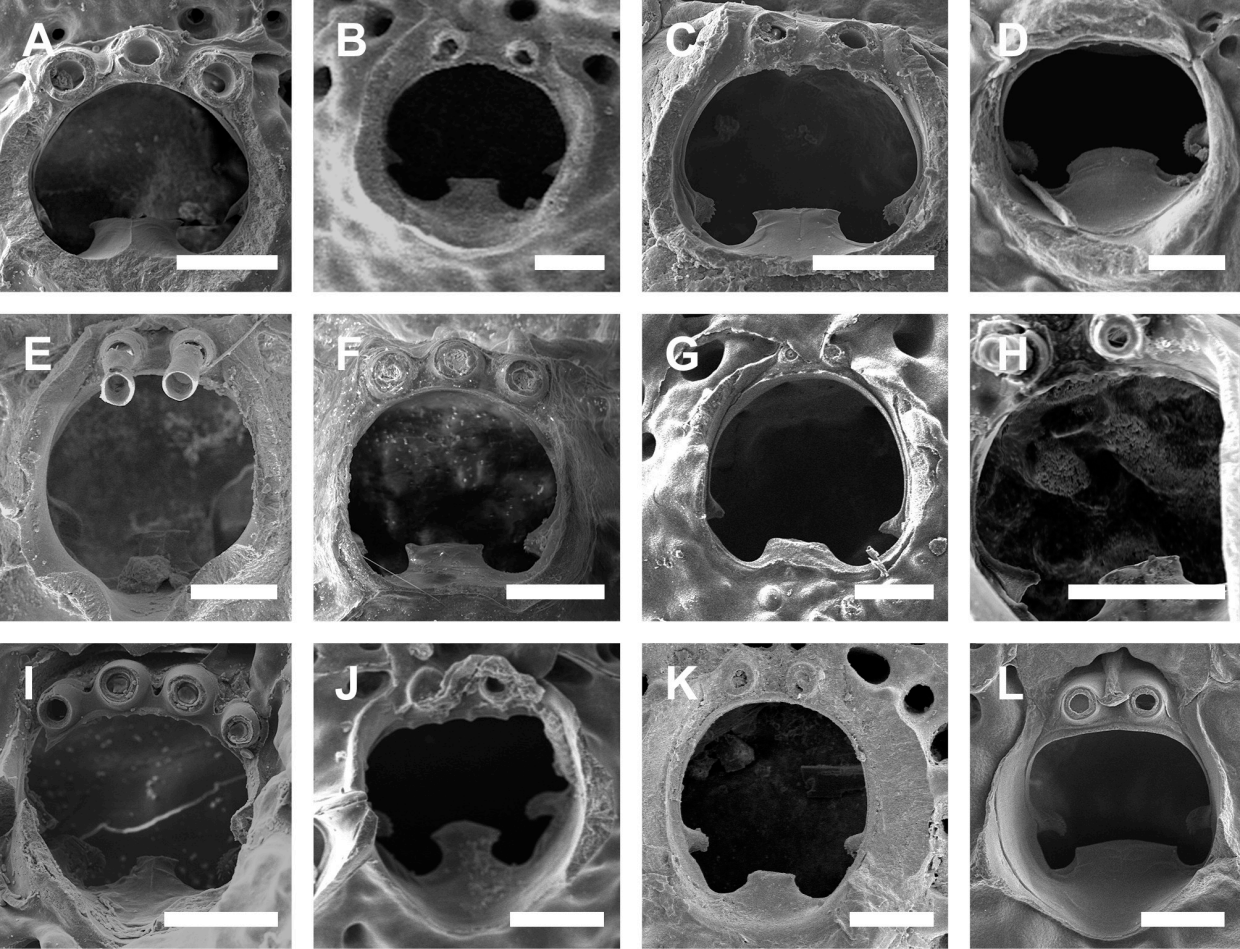
Primary orifices of the studied species. (A) *P*. *abrolhosensis*, (B) *P*. *alba*, (C) *P*. *bimucronata*, (D) *P*. *distincta*, (E) *P*. *dubitata*, (F) *P*. *longirostrata*, (G) *P*. *pinctatae*, (H) *P*. *egyptiaca*, (I) *P*. *serrula*, (J) *P*. *simpulata*, (K) *P*. *winstonae*, (L) *Parasmittina falciformis* sp. nov. Scale bars: 50 μm.

Features of the primary orifice provided the most reliable taxonomic characters to distinguish *Parasmittina* species, as previously pointed out to the Indo-Pacific fauna by Tilbrook [8]. The combination of distal margin and condyle ornamentations, number and profile of spines, and lyrula and orifice width ratio is unique to each species, including in the studied SW Atlantic taxa (Fig 16). Generally, most *Parasmittina* species have a smooth distal margin, 1–4 oral spines, robust and serrated condyles, and a lyrula occupying less than half of the orifice width.

**Fig 17:**
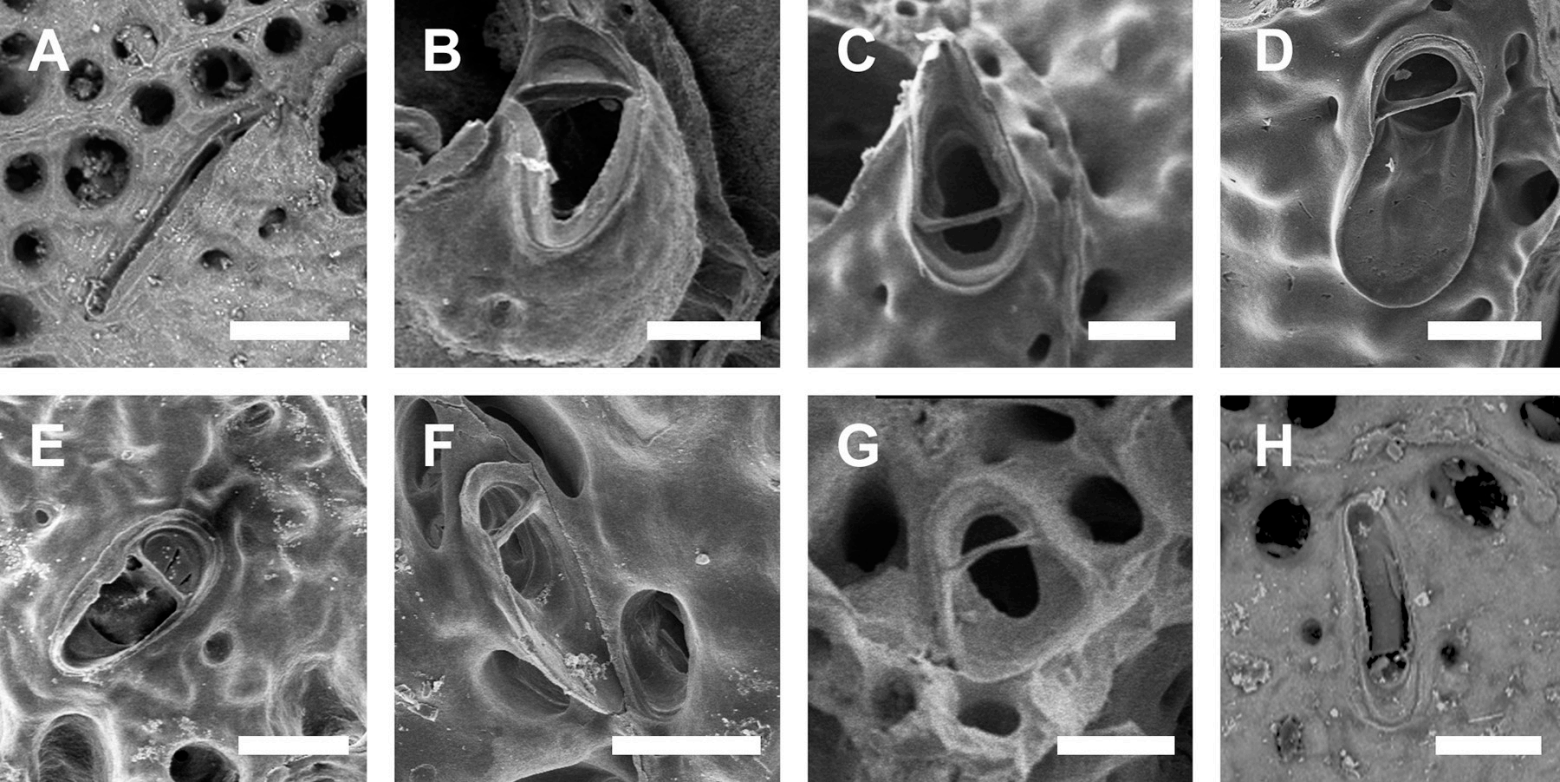
Small avicularia of the studied species. (A–D) latero-oral avicularia, (E–H) marginal/frontal avicularia. (A) elongate as in *P*. *areolata*, (B) subtriangular bulbous as in *P*. *abrolhosensis*, (C) subtriangular as in *P*. *simpulata*, (D) oblong as in *P*. *simpulata*, (E) subtriangular as in *P*. *abrolhosensis*, (F) sublanceolate as in *P*. *pinctatae*, (G) spatulate as in *P*. *winstonae*, (H) elliptical as in *P*. *abrolhosensis*. Scale bars: 50 μm.

**Fig 18:**
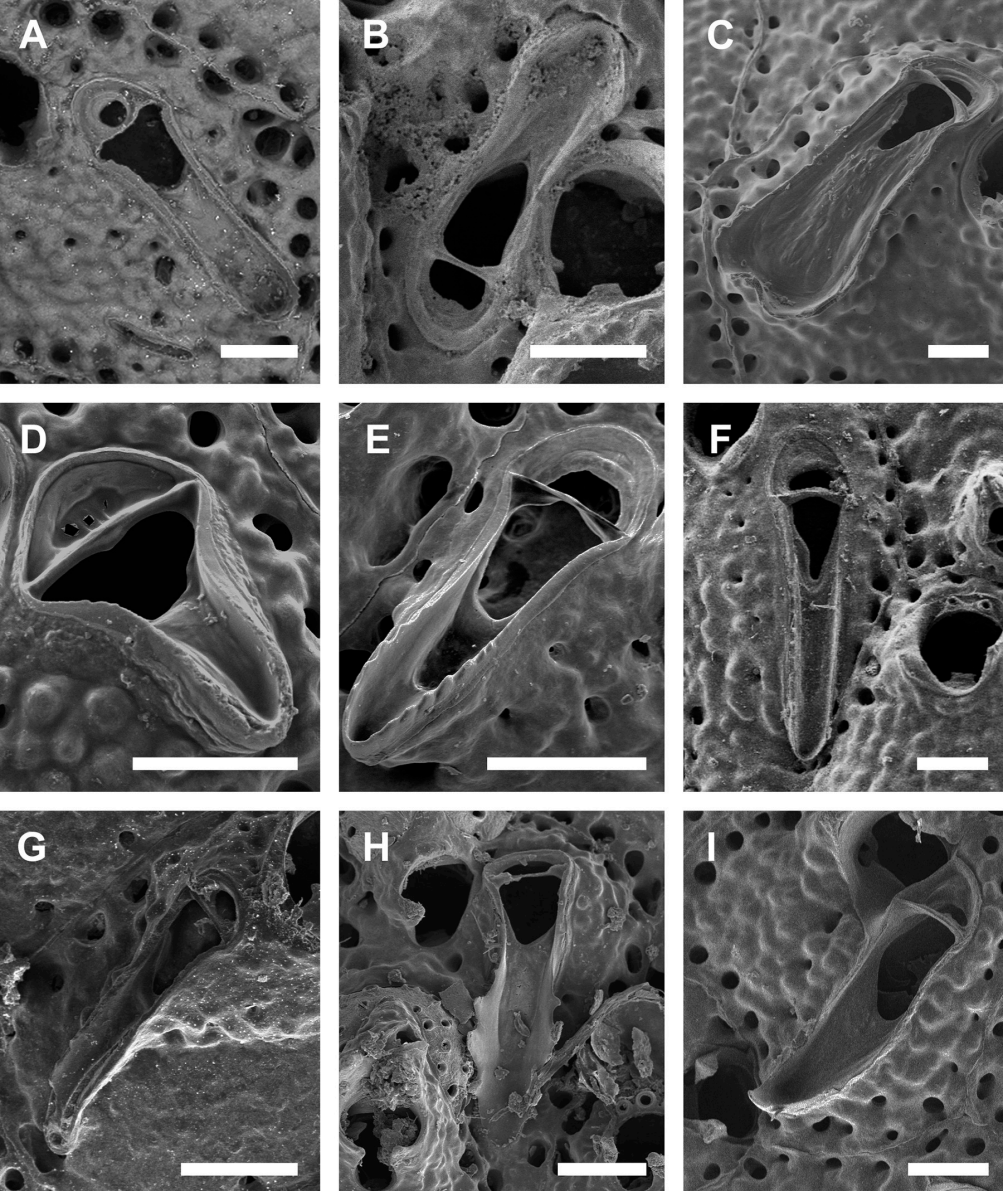
Large avicularia of the studies species. (A) spatulate of *P*. *abrolhosensis*, (B) spatulate of *P*. *winstonae*, (C) spatulate of *P*. *simpulata*, (D) subtriangular of *P*. *dubitada*, (E) subtriangular of *P*. *egyptiaca*, (F) sublanceolate of *P*. *bimucronata*, (G) sublanceolate of *P*. *longirostrata*, (H) noodle tong of *P*. *serrula*, (I) sublanceolate of *Parasmittina falciformis* sp. nov. Scale bars: 100 μm.

Regarding avicularia, the small ones originated from latero-oral pores can be placed disto- and/or proximolaterally to the orifice (Fig 17A–17D), and the small avicularia originated from marginal pores can be placed near zooidal margins and/or proximally in o the frontal wall (Fig 17E–17H). The large avicularium originated from a latero-oral pore and is typically placed proximolaterally to the orifice (Fig 18A–18H).

Avicularia of *Parasmittina* can be characterized in eight main types based on its morphologies: elongate (profile linear and narrow, with parallel margins, Fig 17A); subtriangular (profile subtriangular, with a pointed tip, Fig 17B, 17C, 18D); oblong (profile rectangular, with rounded margins, Fig 17D); sublanceolate (profile spear-shaped, with a slightly curved tip, Fig 17F, 18F, 18I); spatulate (profile spoon-shaped, with distal constriction, Figs 17G, 18B, 18C); elliptical (profile oval-shaped, with no constriction, Fig 17H); noodle tong (profile broadly spatulate, with coarsely serrated margins, Fig 17H), and hatchet-shaped (profile subtriangular, with convex proximal margin, not seen in the studied species).

Most species of *Parasmittina* have one or two morphologies of small avicularia and only one morphology of large avicularia. However, several combinations of avicularia placements and morphologies are seen among taxa, with no apparent pattern correlating these features. For instance, based on the morphology of the small latero-oral avicularia, most *Parasmittina* species can be assigned in two groups–with oblong and/or subtriangular avicularia (as *P*. *bimucronata*, *P*. *egyptiaca* and *P*. *simpulata*) or with elongate avicularia (as *P*. *abrolhosensis*, *P*. *areolata* and *P*. *spathulata)*. Only *P*. *longirostrata* and *P*. *nasuta* have small latero-oral avicularia oblong and/or elongate among all species. Also, the small elongate avicularia are always placed proximolaterally to the orifice, and most taxa with these avicularia lack a distolateral avicularium.

Interestingly, some nominal species can only be distinguished from congeners by characters that are known to differ pending on habitat and colonial development (i.e., [2, 4, 5, 21]), mainly the number of oral spines or absence of avicularia—(*P*. *alba*, *P*. *betamorphaea* and *P*. *lavela*), (*P*. *areolata* and *P*. *trunculata*), (*P*. *amazonensis*, *P*. *fraseri*, *P*. *indiginella* and *P*. *talismani*), (*P*. *barbadensis*, *P*. *parsevaliformis* and *P*. *simpulata*), (*P*. *longirostrata* and *P*. *serruloides*), (*P*. *serrula* and *P*. *luteoserrula*). Thus, these little differences among taxa may represent intraspecific variations rather than different species. Currently, there are no integrative studies (combining morphology and moleculear analysis) to investigate species delimitation within the genus *Parasmittina*. Therefore, further studies including more colonies and other biological data, particularly molecular analysis, are needed to help clarify its taxonomic identity. Thus, to prevent to erect of new names and new synonymies based on characters that can be considered as intraspecific variations, here we choose to discuss and indicate the probable occurrence of species complex in *Parasmittina*, attributing the most appropriate available name to the studied SW Atlantic specimens and attempting to maintain some taxonomic stability in the genus. Analysis including colonies in different astogenetic stages, added with a precise characterization of the primary orifice, avicularia, and ovicells, are strongly required for a reliable taxonomic assignment (i.e., [2, 4, 5, 9, 17, 21, 38]).

**Fig 19:**
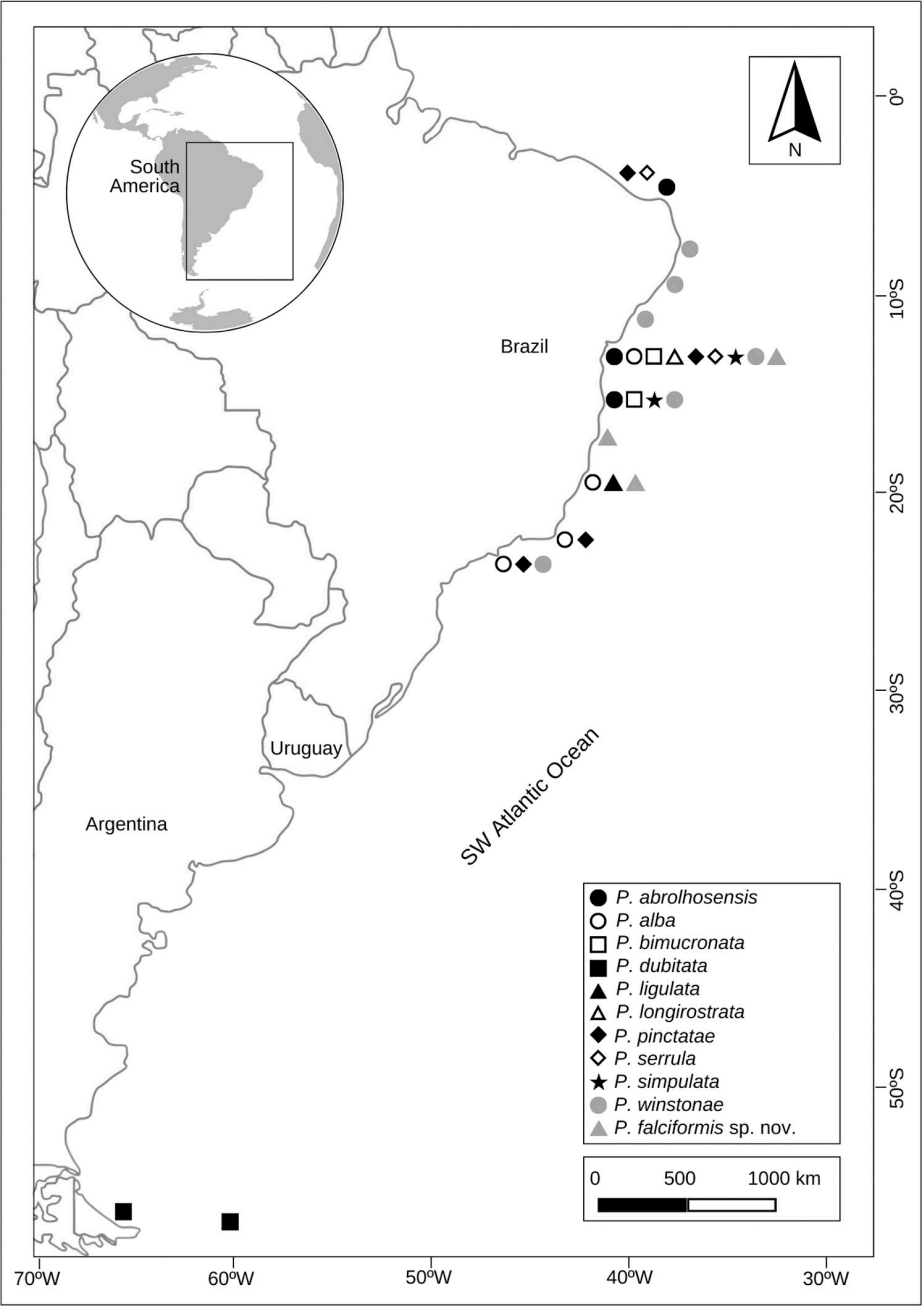
Distribution of *Parasmittina* species studied along the Southwestern Atlantic Ocean. Symbols: black circle, *P*. *abrolhosensis* Ramalho *et al*., 2018; white circle, *P*. *alba* Ramalho, Muricy & Taylor, 2011 [14]; white square, *P*. *bimucronata* (Hincks, 1884b); black square, *P*. *dubitata* Hayward, 1980; black triangle, *P*. *ligulata* comb. nov. (Ridley, 1881); white triangle, *P*. *longirostrata* Liu in Liu, Yin & Ma, 2001; black lozenge, *P*. *pinctatae* Liu in Liu, Yin & Ma, 2001; white lozenge, *P*. *serrula* Soule & Soule, 1973; black star, *P*. *simpulata* Winston, Vieira & Woollacott, 2014; grey circle, *P*. *winstonae* Liu in Liu, Yin & Ma, 2001; grey triangle, *Parasmittina falciformis* sp. nov.

Among the eleven species of *Parasmittina* reported so far from the SW Atlantic, four species—*P*. *betamorphaea*, *P*. *munita*, *P*. *spathulata* and *P*. *trispinosa—*were not recognized in this study (Fig 19).

**Fig 20:**
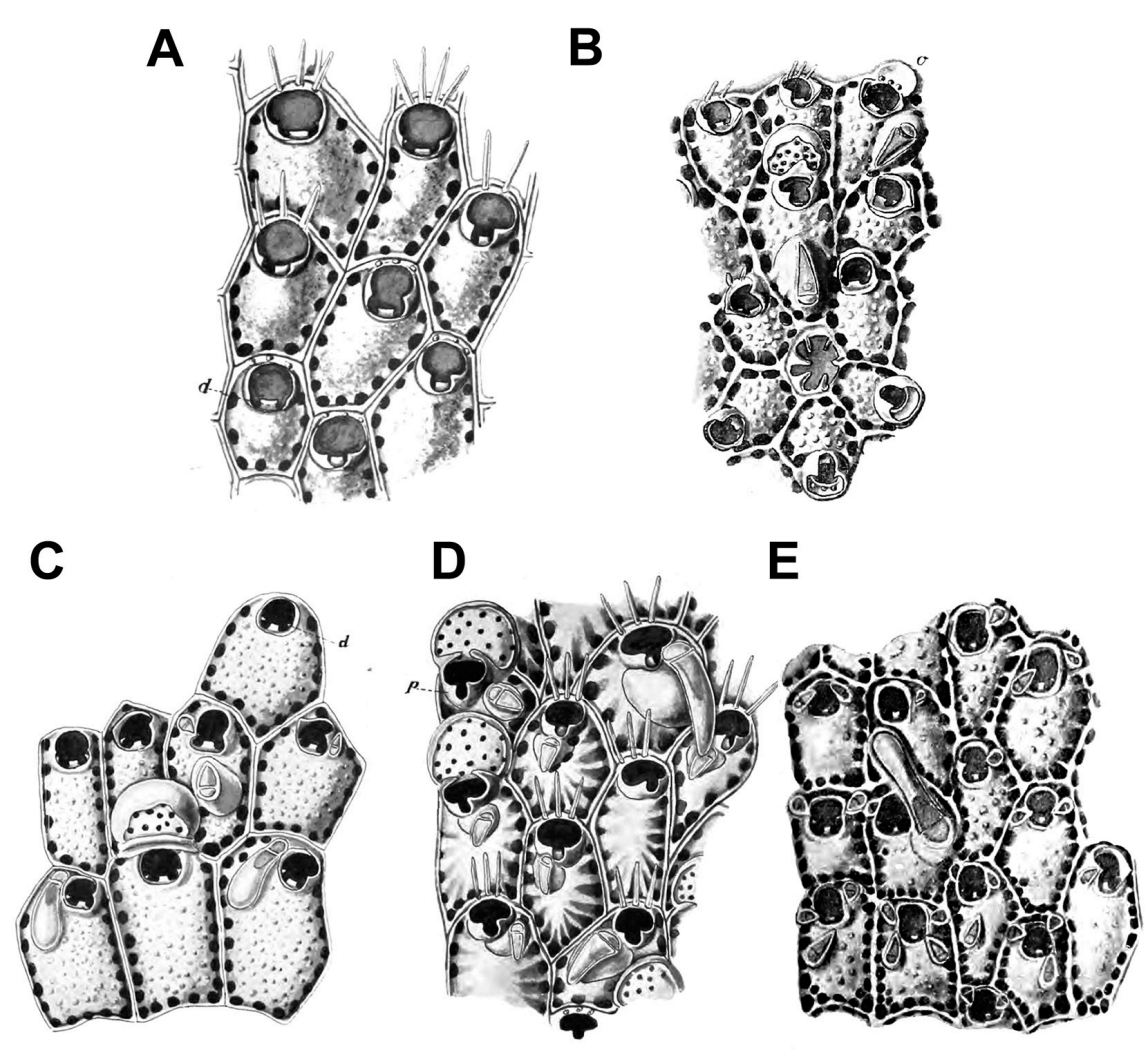
*Parasmittina*. species from Brazil studied by Marcus (1937, 1938, 1939). (A) *Smittina trispinosa*, Marcus, E. det. (1937), (B) *Smittina trispinosa* var. *munita*, Marcus, E. det. (1937) = *P*. *bimucronata* (Hincks, 1884b), (C) *Smittina trispinosa* var. *nitida*, Marcus, E. det. (1937) = *P*. *pinctatae* Liu in Liu, Yin & Ma, 2001, (D) *Smittina trispinosa* var. *munita* Marcus, E. det. (1937) = *P*. sp., (E) *Smittina trispinosa* var. *loxa* Marcus, E. det. (1939) = *P*. *winstonae* Liu in Liu, Yin & Ma, 2001.

Reports of *P*. *betamorphaea* [25], as well as reports of Luederwaldt [53] and Marcus [23] of *P*. *trispinosa*, are here assigned to *P*. *pinctatae*. Part of the records of *P*. *munita* [26] are assigned to *P*. *falciformis* sp. nov., and other records still need review [23, 34] (Fig 20). Finally, reports of *P*. *spathulata* comprise at least two taxa, including *P*. *abrolhosensis* [25, 26, 34] and *P*. *simpulata* [26].

Among species here recognized to occur in Brazil, five were originally described based on specimens from the area (*P*. *abrolhosensis*, *P*. *alba*, *P*. *ligulata* comb. nov., *P*. *simpulata* and *P*. *falciformis* sp. nov.). As mentioned previously, *P*. *alba* and *P*. *simpulata* are part of species complexes that need further investigation. The remaining species described here are mainly known from the Pacific. Due to the unexpected natural occurrence of *P*. *bimucronata*, *P*. *longirostrata*, *P*. *pinctatae*, *P*. *serrula* and *P*. *winstonae* in the SW Atlantic, we evaluated the exotic status of these species in the area (acc. [31]) (Table 11).

**Table 11:** Correspondence of criteria to classify *Parasmittina* species as exotic in the SW Atlantic (acc. Chapman & Carlton, 19991, 1994; Miranda *et al*. 2008; Xavier *et al*. 2021), with the respective number of positive/negative criteria used. 0, criterion not applicable due to lacking data; +, criterion applied positively to exotic status; −, criterion applied negatively to exotic status. N = number of attributes applied positively/negatively. Local criteria: 1 = local appearance where not found previously, 2 = local dispersal after introduction, 3 = association with human mechanisms of dispersal, 4 = prevalence or restriction to new or artificial environment, 5 = restricted distribution when compared to ecologically similar native species. Global criteria: 6 = widespread geographic distribution with isolated populations, 7 = active and passive dispersal mechanisms incapable of achieving the current distribution, 8 = exotic evolutionary origin. Status: E = exotic, C = cryptogenic.

Species	Local criteria					Global criteria			N	Status
	1	2	3	4	5	6	7	8		
*P*. *bimucronata *	−	0	0	−	+	+	+	0	3/2	C
*P*. *longirostrata*	+	0	+	+	+	+	+	0	6/0	E
*P*. *pinctatae*	−	0	+	−	−	+	+	0	3/3	C
*P*. *serrula*	+	0	+	+	+	0	+	0	5/0	C
*P*. *winstonae*	−	0	+	−	−	+	+	0	3/3	C

One species was assigned as to be exotic (*P*. *longirostrata*), and the others were considered cryptogenic, including some (*P*. *bimucronata*, *P*. *pinctatae* and *P*. *winstonae*) previously misassigned to distinct species [10, 11, 12, 26, 53, 58, 60, 61]. These species are widespread along the Brazilian coast, growing on artificial and natural surfaces, indicating that these taxa are well-established in the studied area. Cryptogenic bryozoans must be treated as a cause of concern since, as exotic taxa, they can grow into dense colonies that can influence the associated and native fauna and have economic effects on human activities, including beaches usage, aquaculture, shipping, and fishing (acc. [15, 31, 62]). Thus, more studies with the species complexes recognized here are suggested to determine these taxa origin and help prevent bioinvasion events along the SW Atlantic.

## Supporting information

S1 Table (XLSX)
